# Determining the psychometric properties of a novel questionnaire to measure “preparedness for the future” (Prep FQ)

**DOI:** 10.1186/s12955-021-01759-z

**Published:** 2021-04-15

**Authors:** Daren K. Heyland, J. Paige Pope, Xuran Jiang, Andrew G. Day

**Affiliations:** 1 Department of Critical Care Medicine, Kingston Health Science Centre, Kingston, ON Canada; 2 Clinical Evaluation Research Unit, Kingston Health Science Centre, Kingston, ON Canada; 3 Department of Public Health Sciences, Queen’s University, Kingston, ON K7L 2V7 Canada; 4 Department of Kinesiology and Physical Education, University of Lethbridge, Lethbridge, AB Canada

**Keywords:** Aging, Survey, Psychological health, Health status

## Abstract

**Background:**

People are living longer than ever before. However, with living longer comes increased problems that negatively impact on quality of life and the quality of death. Tools are needed to help individuals assess whether they are practicing the best attitudes and behaviors that are associated with a future long life, high quality of life, high quality of death and a satisfying post-death legacy. The purpose of paper is to describe the process we used to develop a novel questionnaire (“Preparedness for the Future Questionnaire™ or Prep FQ”) and to define its psychometric properties.

**Methods:**

Using a multi-step development procedure, items were generated, for the new questionnaire after which the psychometric properties were tested with a heterogeneous sample of 502 Canadians. Using an online polling panel, respondents were asked to complete demographic questions as well as the Prep-FQ, Global Rating of Life Satisfaction, the Keyes Psychological Well-Being scale and the Short-Form 12.

**Results:**

The final version of the questionnaire contains 34 items in 8 distinct domains (“Medico-legal”, “Social”, “Psychological Well-being”, “Planning”, “Enrichment”, “Positive Health Behaviors”, “Negative Health Behaviors”, and “Late-life Planning”). We observed minimum missing data and good usage of all response options. The average overall Prep FQ score is 51.2 (SD = 13.3). The Cronbach alphas assessing internal reliability for the Prep FQ domains ranged from 0.33 to 0.88. The intra-class correlation coefficient (ICC) used to assess the test–retest reliability had an overall score of 0.87. For the purposes of establishing construct validity, all the pre-specified relationships between Prep FQ and the other questionnaires were met.

**Conclusion:**

Analyses of this novel measure offered support for its face validity, construct validity, test–retest reliability, and internal consistency. With the development of this useful and valid scale, future research can utilize this measure to engage people in the process of comprehensively assessing and improving their state of preparedness for the future, tracking their progress along the way. Ultimately, this program of research aims to improve the quality and quantity of peoples live by helping them ‘think ahead’ and ‘plan ahead’ on the aspects of their daily life that matter to their future.

**Supplementary Information:**

The online version contains supplementary material available at 10.1186/s12955-021-01759-z.

## Background

People are living longer than ever before [1]. However, with living longer comes increased health problems that negatively impact on quality of life [2, 3]. In addition, studies document the financial hardship experienced by many older persons and that many retirees have insufficient funds [4]. And when they cross the finish line, many are poorly prepared for the final stages of life and experience poor quality end of life care [5]. All of this begets; can people do more to prepare to be older?

All of us are in training to become an older person. Perhaps, part of the problem is that we, as a society, do not realize or prioritize the fact that the choices we make today determine how successfully we will age in the future. To the extent that people do think ahead and see themselves as an older person, place some value on that future stage of life, and believe they have ‘control’ over their destiny, they will make better lifestyle choices today to arrive at a better place tomorrow [6]. For example, people in the general population who never smoke, maintain a normal body mass (BMI range of 18.0–24.9), do 30 min or more of vigorous exercise daily, maintain moderate or no alcohol consumption, and have a healthy diet could prolong their life expectancy at 50 by an additional 14.0 and 12.2 years for women and men respectively, compared to controls that did not adopt any of these lifestyle behaviors [7]. These same lifestyle choices also translate into lower chances of developing medical diseases, like diabetes, heart problems, dementia, etc. [8]. The economic consequences of these ‘modifiable’ lifestyle factors are staggering. In the USA, a recent analysis determined that 27% of the annual health care spending was attributable to these five modifiable risk factors [9]. That translate into 730 billion dollars annually spent in managing diseases related to behaviors we have the ability to control. Further, experts suggest that 75% of what determines how well we age is due to lifestyle factors or other factors within our locus of control [10]. So if a person sees themselves in training to become an older person, they are more likely to make better lifestyle choices today that will increase their chances of living longer and *living better*.

We recently surveyed 502 Canadians over the age of 18 and asked them questions about their views on aging (unpublished data from prior sample, see Additional file 1: eTable 1). Whilst the overwhelming majority felt it was important to think about themselves as an older person, few people regularly spend time thinking about what it will be like for them as an older person. When they do, respondents were split whether they saw themselves as an older person in the future in a positive light or a negative one. A significant number of respondents lacked the confidence that they could successfully grow older and many felt it was not up to them, that there were external factors influencing the success of their aging experience. It seems that people need help in ‘thinking ahead’, ‘planning ahead’ and putting themselves in the driver’s as their own locus of control, so as to move forward with confidence in creating a long, high-quality life and high-quality death.

Measurement precedes improvement. If we want to be able to help people better prepare for the future, we need to be able to measure their current ‘state of preparedness for the future.’ The purpose of this paper is to describe the process we used to develop a novel questionnaire (“Preparedness for the Future Questionnaire ™ or Prep FQ”) and to assess its psychometric properties for evaluating a person’s current state of preparedness for their future as an older person. The aim of this questionnaire is to be used to help people think more about their future as an older person and realize the things they could be doing today to age optimally.

## Methods

This project is a multi-phase study aimed at developing and providing initial validation of a novel questionnaire, the Preparedness for the Future Questionnaire ™ (Prep FQ).

### Item generation and refinement

Items for the Prep FQ were generated from three sources. Conceptually, we believe we can improve the health outcomes, quality of life, survival, and end of life experience by helping people think ahead and plan ahead [6]. For example, there is a high level of evidence that current lifestyle behaviors, such as smoking, eating healthy, etc., impact longevity [7] or that planning for your future medical care in advance translates into improved health outcomes for both patients and their substitute decision-makers [11]. Accordingly, we carefully searched the broad scientific literature to identify several key aspects of successful aging, optimal aging, death preparation, end of life, and post-death (legacy contributions). Potential topics were included if a particular activity in the present was shown to impact future quality or quantity of life (such as quitting smoking or healthy eating) or if it was a practical suggestion associated with optimal life and death experiences (legally documenting substitute decision-maker or wills and estate planning, for example). Included topics and evidence supporting their impact are summarized in Additional file 1: eTable 2.

In addition, in 2019, we surveyed a separate cohort of 500 Canadians regarding their views on aging (unpublished data discussed in the introduction). We asked the following open-ended question, “Please describe what activities or behaviors you are currently doing to prepare for a great future as an older person.” Responses were reviewed by the principal author (DKH) to generate items for consideration for the Prep FQ. The responses included actions such as eating healthy, exercising regularly, good sleep habits, saving money, for example. If responses were supported by data and consistent with our conceptual framing, they were considered for inclusion in the questionnaire. Thirty-one items were included in the initial version of the questionnaire.

Each of these potential aspects was then incorporated into an item on the questionnaire. We created response options that reflected the degree of completion or adherence with the related attitude or behaviors listed in the questionnaire. Finally, we piloted an early version of the questionnaire on a group of 20 lay people and health professionals, either individually or in a focus group. We solicited feedback on both the items and response options and whether they had additional items for consideration. Feedback led to further refinement of the items and response options. Three items (Leisure participation, Legacy Planning and Life-long learner) were added to the list as a consequence of this consultation.

In the final version of the questionnaire, we assigned points based on the item’s impact on quality and quantity of life (see Additional file 1: eTable 2). Items that impacted quantity of life were given a weight 3 times more and items that impacted quality of life were weight twice as much compared to just practical suggestions and more points per item were given in the respondent was more compliant with that item. The principal authors (DKH, PP, AD) collaborated and agreed on all point assignments. The “overall” Preparedness score is the sum of points from the responses to all of the answered questions. The domain scores are the sum of points from all answered questions belonging to each domain. The domain scores would have been considered missing if more than half of the responses applicable to the respondent for the domain were missing, but the Qualtrics system did not allow entry of records with any missing data. All scores were re-scaled to range between 0 (worst -lowest possible total points given the applicable answered questions) and 100 (best -highest possible total points given applicable answered questions). Not all questions were applicable to all participants.

### Determining the psychometric properties of the preparedness for the future questionnaire

The validation phase consisted of a cross-sectional survey of 502 Canadians registered with Qualtrics’ online polling panels. To be eligible for this project, panelist had to be living in Canada, speak and read English, and be 18 years of age or older. We strategically sampled 1/3 of participants from each of the following age ranges so we ended up with a representative sample of adults and would be able to compare subgroup differences: 44 years of age or less, 45–65, and 66 years old or older. To obtain a representative sample of Canadians, we aimed to enroll 1/4 from Western provinces, 1/4 from Ontario, 1/4 Quebec; and 1/4 from Atlantic Canada.

In order to test the reproducibility (test–retest reliability) of our novel questionnaire, the Qualtrics staff re-administered the Prep FQ to a subset of 50 participants who are enrolled in the project one week later. Prior to approaching the participants for the reliability assessment, the assistant asked the potential respondent if their life circumstances have changed in the past week. People who said ‘No’ were recruited to this re-test. We justify a one-week period as this is considered sufficient time for the respondents to have forgotten their original answers, but a short enough interval for little change in their lives to have occurred.

At the time of the first online interview, we also collected the following demographic data from participants: age, sex, location, marital status, family circumstances, level of education, language used on a daily basis, global rating of quality of life, and presence of significant health problems. All participants were asked all questions except patients < 60 years old were not asked 7 questions pertaining to physically and social activities, leisure activities, living independently, funeral and burial plans and legacy plans as they were judged to be less relevant to younger people and much of the supportive literature has been conducted in older persons exclusively. In addition, two questions (small business succession planning and family caregiver support) were conditional and not intended to be answered by all respondents.

The primary purpose of this phase was to determine the psychometric properties of the Prep FQ (item response rates, validated domains, internal consistency, reproducibility, and construct validity). We reviewed the response distribution and frequencies, and percent non-response for each item. Items with large amounts of non-response were flagged for potential removal. As a secondary objective, we then conducted an exploratory factor analysis (EFA) to help identify the factor structure of the questionnaire. This EFA guided the combining of items into domains.

With the finalized version of the questionnaire, we used Cronbach’s alpha and McDonald's Omega coefficient scores to evaluate the internal consistency of responses to items from the same domain [12, 13]. As there is no ‘gold standard’ or other validated instruments for measuring future preparedness, we developed a multifaceted approach to validating our novel questionnaire. In the development to date, we utilized a rigorous, comprehensive approach to establishing face and content validity. In this study, we examined construct validity. We expected the finalized version of the questionnaire to be associated with other validated questionnaires measuring potential health and psychological outcomes of someone that is well prepared for the future. Hence, once the questionnaire had been finalized, the Prep FQ domain scores were compared to a single item Global Rating of Life Satisfaction (GRLS), the Psychological Well-Being (PWB) scale and the Short-Form 12 (SF-12), a general status health-related quality of life measure that has 2 summary measures, the Physical Component Summary (PCS) score and the Mental Component Summary Score (MCS).

### Global rating of life satisfaction

Subjective well-being is a high-level concept that captures the affective feelings and cognitive judgments people have about the quality of their lives. Life satisfaction is a component of subjective well-being that focuses on whether one is happy with one’s life. Greater life satisfaction is associated with positive life outcomes, such as health [14], income [15], and better workplace performance [16]. We expect patients who are more prepared for the future to have greater life satisfaction, using this measure as an indication of construct validity. While longer measures to assess life satisfaction exist, a single item, global rating of life satisfaction has been shown to have similar psychometric properties to longer scales and is considered both reliable and valid [17, 18]. Therefore, we asked, “In general, how satisfied are you with your life?” with a 7-point scale from 1 (Completely Dissatisfied) to 7 (Completely Satisfied) used to categorize their answers.

### Psychological well-being scale

Based on an extensive review of the literature, as well as existential and utilitarian philosophy, Ryff [19] defined psychological well-being as a process of self-realization, consisting of six dimensions: autonomy, environmental mastery, personal growth, positive relations with others, purpose in life and self-acceptance [19]. She then created a scale to measure these constructs known as the “Psychological Well-Being Scale” or PWB. This scale has been used widely and has been shown to be reliable, valid, responsive to psychological interventions [20–22]. Domain scores measuring the six dimensions are calculated by averaging the response scores of the seven items from each dimension. We created an overall score by averaging all 42 items. We expected people who score higher on the Prep FQ would have a greater PWB domain scores and people who score higher on the psychology domain of Prep FQ to have even a greater correlation with PWB domain scores compared to the correlation with the overall Prep FQ score.

### Short Form-12

The SF-12v1 is a multipurpose survey of general health status consisting of eight domains that uses just 12 questions to measure functional health and well-being from the patient’s point of view [23]. Taking only two to three minutes to complete, the SF-12v1 covers the same eight health domains as the SF-36v2 with one or two questions per domain and is highly correlated with the summary scores of the SF 36. The SF12v1 has excellent validity, reliability and internal consistency [23]. Given the long duration of the whole question set for participants, we felt the SF-12v1 is a practical, reliable and valid measure of physical and mental health for our purposes. We expected people who score higher on the Prep FQ would have higher SF-12 summary scores and people who score higher on the health domain of Prep FQ to have even a greater correlation with SF-12 Physical component summary scores.

### Construct validity

In summary, a priori, we hypothesize that we would observe weak-to-moderate correlations between these different but related measures. Specifically, we expect the following:

1. Overall Prep FQ score would correlate in a positive direction with all PWB domain and overall scores, GRLS, and SF-12 PCS and SF-12 MCS because someone who is better prepared for the future should enjoy a higher quality of life and life satisfaction. However, we expected these correlations to be weak-moderate and not strong because there are other determinants to these health outcomes than the state of preparedness.
2. We expected the Psychological Well-being domain of the Prep FQ would correlate in a positive direction with PWB domain and overall scores, GRLS, and SF-12 MCS and that these correlations will be greater in magnitude that the correlations observed with the overall Prep FQ because the Prep FQ overall score includes measures unrelated to psychological well-being.
3. We expected the correlation of the Psychological Well-being domain of the Prep FQ to be weakly correlated with the SF-12 PCS because they are measuring 2 different health constructs and that this correlation would be less than the correlations observed in the above 2 analyses.
4. We expected the Positive Health Behavior domain of the Prep FQ will correlate in a positive direction with the SF-12 PCS and that these correlations will be greater in magnitude that the correlations observed with the overall Prep FQ (Analysis #1) because of the tight relationship of the 2 measures of physical behavior and health in contrast to the overall score, which includes unrelated measures.

In addition, to further add to the validity of the questionnaire, we examined the Prep FQ scores in various subgroups to demonstrate the ability of the novel questionnaire to discriminate different states. Specifically, we expected to see higher scores in the Medico-legal domains in people who were married and with children and lower scores in the Positive health Behavior domains in people with chronic health conditions.

### Sample size and justification

We planned to enroll 500 participants so that the average width from the lower to upper 95% confidence limits for Pearson correlations of 0.3, 0.5 and 0.8 would be 0.16, 0.13 and 0.06 respectively, and for Cronbach Alpha’s of 0.5, 0.7 and 0.9 assuming at least 3 items would be 0.15, 0.09 and 0.03 respectively. We enrolled a sub-sample of 50 participants for the test–retest reliability assessment so the average one-sided lower 95% confidence limit for ICCs of 0.7, 0.8, and 0.9 would be 0.56, 0.70 and 0.84.

### Statistical methods

Staff at Qualtrics were responsible for data collection and delivery of de-identified data via a secure method to the Clinical Evaluation Research Unit (CERU) at the Kingston General Hospital who were responsible for the analysis. Descriptive statistics were used to describe the responses to all questionnaires.

We used Cronbach’s alpha to measure the internal consistency of the items within each domain [12]. Separate Cronbach’s alphas were calculated for the various participant subgroups based on age (patients ≥ 60 years and < 60 years) and the numbers of conditional questions answered so that the final domains scores could be assessed for each participant subgroup. We also calculated the McDonald's Omega coefficient scores as well which takes into account the strength of association.

between items and constructs and the item-specific measurement errors [13].

We assessed the reproducibility of our novel questionnaire over a one-week period using Intraclass correlation coefficients (ICC) calculated from the one-way Analysis of Variance. For each domain and the overall score we report he ICC with 95% confidence intervals. The ICC is the proportion of the total variance between assessments that is due to difference between respondents rather than differences between the two assessments within the same respondent [24]. ICC values above 0.7 are generally considered good to excellent. Pearson’s correlation coefficient between the Prep PQ domains and other instruments were used as described in the prior section to assess construct validity.

As a secondary analysis, we used exploratory factor analysis (EFA) to guide the grouping of items into domains. The EFA used the common factor model. Since the responses were not normally distributed and some domains were expected a-prior to be correlated, we used iterated principal factor analysis with the oblique PROMAX rotation [25]. Although face validity was considered when loadings were equivocal, items were generally assigned to the factor to which they loaded most heavily. The main EFA only considered the 25 items that were applicable to all participants so that the full sample could be used. We conducted separate EFAs on the common data set (25 items) for participants aged < 60 and 60 and over. We considered item weightings and clinical sensibility in determining the final factor structure. Based on face validity, the 2 conditional questions were assigned to the ‘Planning’ domain and the remaining 7 items only answered by older cohort were combined to form a ‘Late-life Planning’ domain. Domain scores were calculated for each respondent by summing the points to the questions applicable to them. The domain was then linearly rescaled so that 0 and 100 were the worst and best possible score given the items applicable to the respondent.

Finally, we also report the Kaiser–Meyer–Olkin (KMO) measure and Bartlett test of sphericity to judge the suitability of conducting the factor analyses. It is suggested that KOM measure of below 0.50 is unacceptable; > 0.60 is tolerable, overall KMO should be greater than 0.80 [26]. With the Bartlett test of sphericity, a small *p* value (*p* < 0.05) indicates rejecting the null hypothesis which suggest that the data are appropriate for factor analysis [26].

All analysis were done using SAS Version 9.4 (SAS Institute Inc., Cary, NC, USA). We obtained Research Ethics Board approval from Queen’s University. Given this project used de-identified responses from panelists that had consented to participate in Qualtrics survey work, our ethics board waived the need for us to obtain informed consent from study subjects.

## Results

Five hundred and two participants completed all questionnaires. Table 1 presents demographics data on all participants. The average age was 53 ± 17.5 (SD) with 218 (43%) of the sample with an age 60 or more. There were slightly more females (58%) compared to males (42%).

**Table 1:** Demographics by age groups

	All respondents (n = 502)	Respondents age ≥ 60 (n = 218)	Respondents age < 60 (n = 284)	*p* values
Age mean ± SD (range)	53.0 ± 17.5 (19.0–87.0)	69.4 ± 5.5 (60.0–87.0)	40.4 ± 12.3 (19.0–59.0)	< .001
Gender				0.10
Male	210 (41.8%)	103 (47.2%)	107 (37.7%)	
Female	289 (57.6%)	114 (52.3%)	175 (61.6%)	
Other	3 (0.6%)	1 (0.5%)	2 (0.7%)	
Province				< 0.001
Alberta	47 (9.4%)	23 (10.6%)	24 (8.5%)	
British Columbia	50 (10.0%)	28 (12.8%)	22 (7.7%)	
Manitoba	12 (2.4%)	10 (4.6%)	2 (0.7%)	
New Brunswick	27 (5.4%)	3 (1.4%)	24 (8.5%)	
Newfoundland and Labrador	20 (4.0%)	3 (1.4%)	17 (6.0%)	
Northwest Territories	1 (0.2%)	0 (0.0%)	1 (0.4%)	
Nova Scotia	71 (14.1%)	19 (8.7%)	52 (18.3%)	
Ontario	125 (24.9%)	77 (35.3%)	48 (16.9%)	
Prince Edward Island	10 (2.0%)	4 (1.8%)	6 (2.1%)	
Quebec	125 (24.9%)	39 (17.9%)	86 (30.3%)	
Saskatchewan	14 (2.8%)	12 (5.5%)	2 (0.7%)	
Current marital status				< 0.001
Married	221 (44.0%)	114 (52.3%)	107 (37.7%)	
Living as married/common law	44 (8.8%)	8 (3.7%)	36 (12.7%)	
Widowed	32 (6.4%)	26 (11.9%)	6 (2.1%)	
Single	139 (27.7%)	24 (11.0%)	115 (40.5%)	
Divorced or separated	66 (13.1%)	46 (21.1%)	20 (7.0%)	
Do you have any children?				< 0.001
No	256 (51.0%)	98 (45.0%)	158 (55.6%)	
Yes, only 1	89 (17.7%)	32 (14.7%)	57 (20.1%)	
Yes, more than 1	157 (31.3%)	88 (40.4%)	69 (24.3%)	
Highest level of education				0.03
Did not complete secondary school or high school	12 (2.4%)	8 (3.7%)	4 (1.4%)	
Completed secondary or high school	107 (21.3%)	50 (22.9%)	57 (20.1%)	
Had some university education or completed	197 (39.2%)	94 (43.1%)	103 (36.3%)	
University degree	135 (26.9%)	44 (20.2%)	91 (32.0%)	
Graduate degree	51 (10.2%)	22 (10.1%)	29 (10.2%)	
Which language do you speak on a daily basis?				0.009
English	415 (82.7%)	193 (88.5%)	222 (78.2%)	
French	75 (14.9%)	21 (9.6%)	54 (19.0%)	
Other (specify)	12 (2.4%)	4 (1.8%)	8 (2.8%)	
In general would you say your physical health is				0.001
Excellent	47 (9.4%)	9 (4.1%)	38 (13.4%)	
Very good	147 (29.3%)	59 (27.1%)	88 (31.0%)	
Good	183 (36.5%)	85 (39.0%)	98 (34.5%)	
Fair	104 (20.7%)	57 (26.1%)	47 (16.5%)	
Poor	21 (4.2%)	8 (3.7%)	13 (4.6%)	
In general would you say your mental health is				0.009
Excellent	97 (19.3%)	49 (22.5%)	48 (16.9%)	
Very good	155 (30.9%)	74 (33.9%)	81 (28.5%)	
Good	160 (31.9%)	71 (32.6%)	89 (31.3%)	
Fair	70 (13.9%)	19 (8.7%)	51 (18.0%)	
Poor	20 (4.0%)	5 (2.3%)	15 (5.3%)	
Do you have				< 0.001
Significant or major chronic health problems	93 (18.5%)	58 (26.6%)	35 (12.3%)	
Few or minor chronic health problems	166 (33.1%)	92 (42.2%)	74 (26.1%)	
No chronic health problems	243 (48.4%)	68 (31.2%)	175 (61.6%)	

The final version of the questionnaire contains 34 items in 8 distinct domains (“Medico-legal”, “Social”, “Psychological Well-being”, “Planning”, “Enrichment”, “Positive Health Behaviors”, “Negative Health Behaviors”, and “Late-life Planning”). The “Late-life Planning domain contained 7 items that were only answered by participants aged 60 or older. The raw response frequencies to the numbered items of the Prep FQ are shown in Additional file 1: eTable 3. There were no missing data because the Qualtrics system required a response to all items. The highest non-response (i.e. responded “prefer not to say/answer”) rate for any Prep FQ question was 3% for question 25 “Do you have adequate insurance.”

Based on our prior expectations and the results of the EFA with items answered by all respondents, we selected a 7-factor structure (see Table 2). These 7 factors were named, “Medico-legal”, “Social”, “Psychological Well-being”, “Planning”, “Enrichment”, “Positive Health Behaviors” and “Negative Health Behaviors.” All items loaded with coefficients > 0.30 and ranged from 0.33 to 0.80. KMO and Bartlett test indicate that the data was suitable for factor analysis (see legend of Table 2). However, when considering the secondary EFAs in participants younger and older than 60, considerable differences in item loadings were apparent (see Additional file 1: eTable 4a, b). These differences affect 8 items summarized in Additional file 1: eTable 4c. Additionally, ‘maintaining an optimal BMI’ did not load adequately on any factor in either of these EFAs and ‘smoking’ did load on any factor in the EFA in participants ≥ 60 years.

**Table 2:**
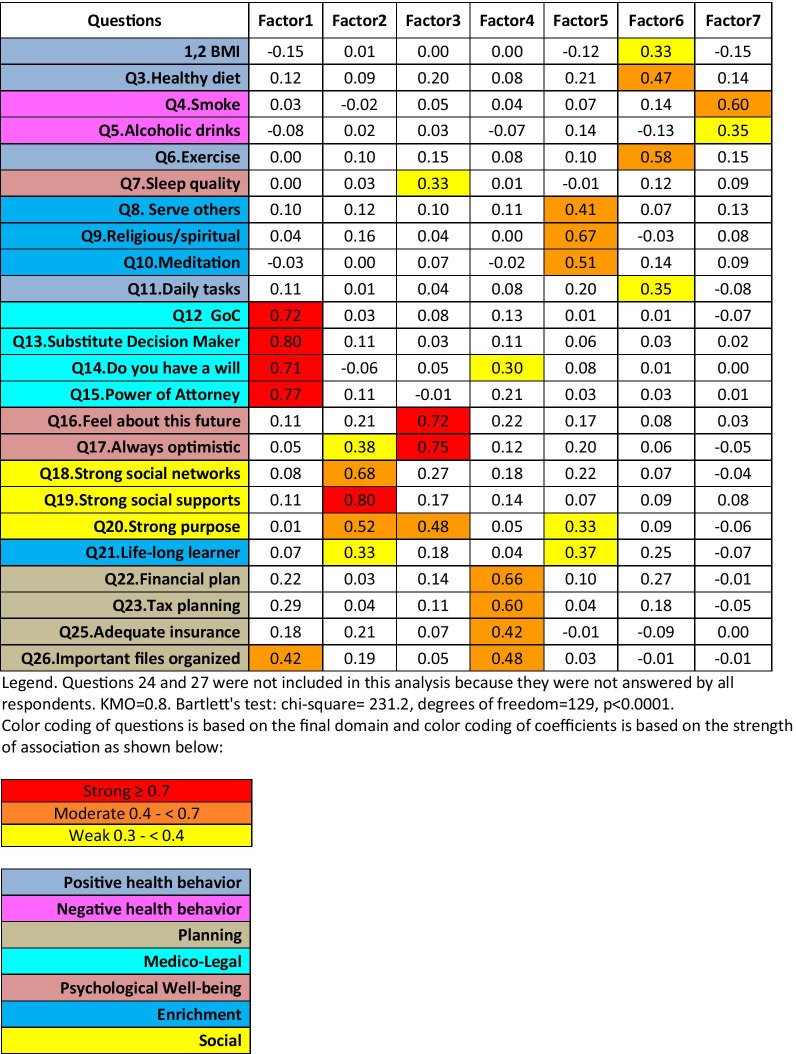
Results of exploratory factor analysis: 7 factor model (n = 502)

Average Prep FQ domain scores range from 27.6 for the Medico-Legal domain to 64.0 for the Negative Health Behavior domain. The average overall Pre FQ score is 51.2 (SD = 13.3).

The Cronbach alphas and McDonald’s Omega coefficient assessing internal reliability for the Prep FQ domains are shown on Additional file 1: eTable 5. The Cronbach alphas value were always close to the McDonald’s Omega coefficient. Their lowest values were both 0.33 for Negative Health Behaviors (2 items) and their second lowest values were 0.50 and 0.53 respectively for Positive Health Behaviors (4 items). Both scores were at least 0.60 all other domains. The intra-class correlation coefficient (ICC) used to assess the test–retest reliability of the Prep FQ domains ranged from 0.69 for the Late-life Planning and Social domains to 0.85 for the Enrichment domain which indicate good to excellent reliability (See Table 3). The overall score had a reliability of 0.87. The Cronbach’s alpha values for the other questionnaires used in this study are shown in Additional file 1: eTable 6.

**Table 3:** Test–retest reliability

Domain	ICC (95% confidence intervals)
Positive health behaviors	0.83 (0.72, 0.90)
Negative health behaviors	0.82 (0.71, 0.89)
Planning	0.81 (0.69, 0.89)
Medico-legal	0.84 (0.74, 0.91)
Psychological well-being	0.84 (0.74, 0.91)
Enrichment	0.85 (0.75, 0.91)
Late-life planning	0.69 (0.46, 0.83)
Social	0.69 (0.51, 0.81)
Total score	0.87 (0.78, 0.92)

Table 4 shows that the Prep FQ met all the pre-specified relationships with the other questionnaires as stated a priori for the purposes of establishing construct validity (see Additional file 1: eTable 7 for complete results of correlations between Prep FQ domains and other questionnaires). Specifically, the overall Prep FQ score was weak to moderately correlated in a positive direction with GRLS, SF-12 PCS, SF-12 MCS and all PWB domains. A one standard deviation increase in the overall Prep FQ score was associated with a one-half standard deviation increase in the GRLS and overall PWB scores and vice versa. In addition, we observed that the Psychological Well-being domain of the Prep FQ was also correlated in a positive direction with GRLS, SF-12 MCS, and all domains of the PWB and that these correlations were greater in magnitude than the correlations observed with the overall Prep FQ except PWB Personal Growth domain. Also, we observed that the Psychological Well-being domain of the Prep FQ was weakly correlated with the SF-12 PCS and that this correlation was less than the correlations observed in the above 2 analyses. Finally, the Positive Health Behaviors domain of the Prep FQ did correlate in a positive direction with the SF-12 PCS and this correlation was greater in magnitude that the correlations observed with the overall Prep FQ.

**Table 4:** Construct validity

Construct assessed	GRLS	Physical component summary score (PCS)	Mental component summary score (MCS)	PWB overall score	PWB autonomy	PWB environmental mastery	PWB personal growth	PWB positive relations	PWB purpose in life	PWB self-acceptance
1. Overall prep FQ score	0.53	0.17	0.35	0.54	0.24	0.44	0.48	0.47	0.51	0.49
2 and 3. Psychological Well-being domains of the prep FQ	0.68	0.22	0.51	0.60	0.32	0.52	0.46	0.47	0.55	0.63
4. Positive health behaviors domains of the prep FQ		0.29								

Estimates are Pearson’s correlation coefficients

In subgroup analysis, people who were married had a higher Prep FQ Medico-legal domain score compared to people who were not married (35.4 vs. 21.6, *p* < 0.001). People with more than one child also had a higher Medico-legal domain score than those with one child or without children (35.1 vs. 22.7 vs. 28.7, *p* < 0.001). Finally, people with some chronic health problems had a lower Positive Health Behavior score compared to those who did not have any chronic health problems (48.3 vs 55.2, *p* < 0.001).

## Discussion

We set out to develop and validate a novel questionnaire to enable self-assessment of individuals contemplating their future as an older person. We derived our items from content analysis of lay respondents, the scientific literature and focus groups with experts and lay representatives. We supported our inclusion of various items referencing the corresponding published evidence. Based on the development methods, we concluded that our questionnaire has face and content validity. In this cross-sectional survey, we collected responses on 502 respondents across Canada and demonstrated good utility (limited “preferred not to say’ and use of full range of responses), validated domain structures and scores, and good test-test reliability. The internal reliability was acceptable for most domains, but was particularly low for the “negative health behaviors” domain which included an item for tobacco use and an item for alcohol consumption. Although there was not a strong response correlation between these two items, we believe that the literature clearly supports that tobacco use and excessive alcohol use are both important negative health behaviors. Thus, we decided to keep these two items in the same domain.

This multi-dimensional questionnaire differs from existing validated questionnaires, such as GRLS, SF-12, or PWB scale, in that it attempts to measure all attitudes, behaviors, and key practices that portend for a longer, higher quality life, high quality death, and positive legacy experience. As such, we did not expect it to correlate highly with questionnaires that measure one aspect of the human experience- life satisfaction, health status, or psychological well-being, for example. Conceptually, we considered these latter measures as related but distinct from Prep FQ and as possible outcome measures. In other words, if a person is highly engaged in thinking about and preparing for a high-quality future, they should have higher scores in these outcome measures, which is what we observed. Practically, then, the Prep FQ can be used as a diagnostic test or self-assessment questionnaire, to help the respondent evaluate where they are at in their ‘readiness for the future.’ Their Prep FQ score, particularly when bench-marked to peers, may serve to motivate individuals to engage more in preparing for the future. Scores on individual items and domains will give individuals a sense of areas of improvement to have a more successful aging experience. We further observed that a one standard deviation increase in the overall Prep FQ score (13 points) was associated with a one-half standard deviation increase in the GRLS and overall PWB scores. By improving a few points on each of the 5 major lifestyle factors and/or engaging in advance care planning, financial planning, legal planning etc., people can easily improve their scores and state of preparedness and this will translate in clinically important and moderately large increases in their life satisfaction, health status and psychological well-being. Given historical high levels of mental illness, low levels of psychological well-being, and an epidemic of obesity and high prevalence of chronic, non-communicable diseases [27, 28], it would be important to widely disseminate tools that help individuals assess and self-manage their health and well-being. This novel self-assessment questionnaire begins to move people in that direction.

The strengths of this work are the robust approach to the development and evaluation of this novel questionnaire including a rigorous sampling method and questionnaire administration that results in a nationally representative sample with no missing data. The weakness of this work include: (1) We could not perform a systematic review to identify all topics that impact quality and quantity of life, quality of death and legacy experience as the topics were too broad and some (such as most of the planning topics), did not have an evidentiary basis to support their impact. Consequently, we cannot be absolutely sure that we did not miss some important aspect of life and death that belongs in this questionnaire. Having said that, we reached saturation with our existing searches and consultations with over 500 individuals, we are confident that all major aspects are included in this version of the questionnaire. (2) Whilst our sample was representative of people across Canada and reflected both the age and gender mix across Canada, our findings may not be generalizable to minority groups, people of low socio-economic status, or non-English speakers. Future work with this questionnaire can explore the adaptability and utility in these subgroups. (3) Some of the psychometric properties related to individual domain scores, such as the Cronbach’s alpha and McDonald’s Omega coefficient of the Negative Health Behaviors did not meet minimal threshold standards. While low Cronbach alpha’s were not ideal, they were not surprising either, given that each items contained within the Prep FQ represents a unique behavior or attitude. However, the potential alternative of adding items (and in turn participant burden) to increase the internal consistency of the domains is of little value to the ultimate purpose of this scale. We tried to develop the most parsimonious scale that identified key behaviors and attitudes associated with a better future that can be used for individuals to identify their personal opportunities for improvement, and therefore it is the single item that informs the decision rather than the domain. This methodological weakness is only relevant to the domain scores which are less actionable at an individual level. We further acknowledge that the domains suggested by our exploratory factor analyses require further evaluation in a subsequent sample with confirmatory factor analyses. (4) Because some questions did not pertain to all ages, our EFA was repeated separately in participants < 60 and 60 or older. As a consequence, we saw a limited number of items that did not fall into the same age specific domain structure as the overall EFA. These findings question the legitimacy of the domain scores used in a heterogenous sample, but if the questionnaire is principally used as it was intended as a tool for self-assessment and improvement where the individual item and overall score are all that is required, this is not a major impediment to its use. Alternately, having separate questionnaires for younger and older populations may be a barrier to uptake and further development.

## Conclusions

This work represents the development and evaluation of a unique questionnaire to measure the state of people’s preparedness for their life as an older person. Now that we can measure this construct in a reliable and valid way, we can begin to engage people in the process of improving their state of preparedness for the future and will have a psychometrically sound measure to monitor progress of individuals or groups of individuals. Future work will need to establish the ‘predictive validity’ of this questionnaire (that it identifies people that will have poor health or well-being in the future) and its responsiveness (that it changes subsequent to lifestyle interventions). Ultimately, this program of research aims to improve the quality and quantity of peoples live by helping them ‘think ahead’ and ‘plan ahead’ and take control of the things that matter for their future.

## Supplementary Information

**Additional file 1.** Additional information and secondary results related to the psychometric evaluation of the Preparedness for the Future Questionnaire.

## Data Availability

All data generated or analysed during this study are included in this published article and its supplementary information files.
