# Supplementary material for: Determining the psychometric properties of a novel questionnaire to measure “preparedness for the future” (Prep FQ)
Source: Health Qual Life Outcomes. 2021 Apr 15;19:122. doi: 10.1186/s12955-021-01759-z (PMC8048271; doi:10.1186/s12955-021-01759-z)
Supplement: Supplementary file 1 — Additional file 1. Additional information and secondary results related to the psychometric evaluation of the Preparedness for the Future Questionnaire. [file 12955_2021_1759_MOESM1_ESM.docx]

Supplementary Appendix

**eTable 1 – Survey of thoughts and attitudes towards getting older**

|  | **All respondents (n=502*)** | **Age <40 (n=139)** | **Age 40-<60 (n=168)** | **Age 60-<80 (n=142)** | **Age≥80 (n=50)** |
| --- | --- | --- | --- | --- | --- |
| **1. How often do you think about what you will be like when you are older, say 20-30 years or more from now?** |  |  |  |  |  |
| *Never* | 37 (7.4%) | 6 (4.3%) | 6 (3.6%) | 11 (7.7%) | 14 (28.0%) |
| *Seldom* | 126 (25.1%) | 41 (29.5%) | 22 (13.1%) | 47 (33.1%) | 14 (28.0%) |
| *Sometimes* | 226 (45.0%) | 65 (46.8%) | 98 (58.3%) | 47 (33.1%) | 15 (30.0%) |
| *Often* | 99 (19.7%) | 24 (17.3%) | 37 (22.0%) | 34 (23.9%) | 4 (8.0%) |
| *Always* | 14 (2.8%) | 3 (2.2%) | 5 (3.0%) | 3 (2.1%) | 3 (6.0%) |
| **2. When you think about your future as an older person, do you feel positive or negative about this future?** |  |  |  |  |  |
| *Very Positive* | 48 (9.6%) | 13 (9.4%) | 11 (6.5%) | 15 (10.6%) | 9 (18.0%) |
| *Somewhat positive* | 161 (32.1%) | 43 (30.9%) | 51 (30.4%) | 43 (30.3%) | 23 (46.0%) |
| *Neither positive or negative* | 135 (26.9%) | 35 (25.2%) | 48 (28.6%) | 42 (29.6%) | 8 (16.0%) |
| *Somewhat negative* | 126 (25.1%) | 44 (31.7%) | 40 (23.8%) | 32 (22.5%) | 10 (20.0%) |
| *Very Negative* | 32 (6.4%) | 4 (2.9%) | 18 (10.7%) | 10 (7.0%) | 0 (0.0%) |
| **3. How important do you think it is to take time to think ahead about your future as an older person?** |  |  |  |  |  |
| *Extremely Important* | 106 (21.1%) | 30 (21.6%) | 43 (25.6%) | 25 (17.6%) | 8 (16.0%) |
| *Very Important* | 157 (31.3%) | 45 (32.4%) | 50 (29.8%) | 42 (29.6%) | 18 (36.0%) |
| *Important* | 148 (29.5%) | 41 (29.5%) | 48 (28.6%) | 47 (33.1%) | 12 (24.0%) |
| *Somewhat important* | 76 (15.1%) | 19 (13.7%) | 22 (13.1%) | 23 (16.2%) | 11 (22.0%) |
| *Not important* | 15 (3.0%) | 4 (2.9%) | 5 (3.0%) | 5 (3.5%) | 1 (2.0%) |
| **4. I am confident that I can successfully grow older.** |  |  |  |  |  |
| *Agree Strongly* | 97 (19.3%) | 28 (20.1%) | 27 (16.1%) | 21 (14.8%) | 21 (42.0%) |
| *Agree Moderately* | 126 (25.1%) | 27 (19.4%) | 41 (24.4%) | 43 (30.3%) | 14 (28.0%) |
| *Agree Somewhat* | 143 (28.5%) | 49 (35.3%) | 51 (30.4%) | 35 (24.6%) | 8 (16.0%) |
| *Neutral or Undecided* | 70 (13.9%) | 21 (15.1%) | 19 (11.3%) | 24 (16.9%) | 5 (10.0%) |
| *Disagree Somewhat* | 35 (7.0%) | 9 (6.5%) | 12 (7.1%) | 12 (8.5%) | 1 (2.0%) |
| *Disagree Moderately* | 15 (3.0%) | 2 (1.4%) | 7 (4.2%) | 6 (4.2%) | 0 (0.0%) |
| *Disagree Strongly* | 13 (2.6%) | 2 (1.4%) | 11 (6.5%) | 0 (0.0%) | 0 (0.0%) |
| *Prefer not to say* | 3 (0.6%) | 1 (0.7%) | 0 (0.0%) | 1 (0.7%) | 1 (2.0%) |
| **5. Whether I grow old successfully is not entirely up to me.** |  |  |  |  |  |
| *Agree Strongly* | 66 (13.1%) | 13 (9.4%) | 26 (15.5%) | 17 (12.0%) | 10 (20.0%) |
| *Agree Moderately* | 92 (18.3%) | 21 (15.1%) | 26 (15.5%) | 28 (19.7%) | 15 (30.0%) |
| *Agree Somewhat* | 173 (34.5%) | 50 (36.0%) | 55 (32.7%) | 53 (37.3%) | 14 (28.0%) |
| *Neutral or Undecided* | 53 (10.6%) | 15 (10.8%) | 12 (7.1%) | 22 (15.5%) | 4 (8.0%) |
| *Disagree Somewhat* | 65 (12.9%) | 18 (12.9%) | 30 (17.9%) | 12 (8.5%) | 5 (10.0%) |
| *Disagree Moderately* | 29 (5.8%) | 13 (9.4%) | 12 (7.1%) | 3 (2.1%) | 1 (2.0%) |
| *Disagree Strongly* | 22 (4.4%) | 8 (5.8%) | 7 (4.2%) | 6 (4.2%) | 1 (2.0%) |
| *Prefer not to say* | 2 (0.4%) | 1 (0.7%) | 0 (0.0%) | 1 (0.7%) | 0 (0.0%) |

These results are from a distinct dataset done the year previously (unpublished), not from the dataset reported on in this paper. It is a coincidence that the sample size of the prior and current sample were both 502 even though in both cases the target sample size was 500.

*The age groups add up to 499, because in this prior sample, age was missing for 3 respondents.

**eTable 2 – Summary of Supportive Evidence for Included Items**

| **Item** | **Evidence for impact on survival** | **Evidence for impact on quality of life or functional status** |
| --- | --- | --- |
| ***Category 3*** |  |  |
| **Maintaining a normal BMI** | Having an elevated BMI will significantly increase risk of death^^[[1]](#endnote-2)^^ whereas maintaining a normal BMI (<25) is associated with enjoying the greatest number of disease-free years of life.^^[[2]](#endnote-3)^^ | People with an elevated BMI are at increased risk of developing numerous chronic diseases, frailty and cognitive decline including dementia.^^[[3]](#endnote-4)^,^^^[[4]](#endnote-5)^,^[[5]](#endnote-6)^,^[[6]](#endnote-7)^,^[[7]](#endnote-8)^,^^^[[8]](#endnote-9)^^ |
| **Healthy Eating** | People who are more adherent to dietary guidelines and eat healthier are much less likely to develop heart problems and much more likely to live longer.^1,4,^[[9]](#endnote-10)^^ | Poor overall diet quality may increase the risk of becoming frail in old age.^^[[10]](#endnote-11)^^ |
| **Smoking** | Cigarette smoking is the leading preventable cause of mortality.^^[[11]](#endnote-12)^^ Smokers who quit smoking can reduce their risk of developing and dying from tobacco-related diseases, such as lung cancer and other cancers and thereby add years to life.^^[[12]](#endnote-13)^^ | Quitting smoking can reduce the incidence of hip fractures and reduce the incidence of cognitive decline and dementia.^8,^[[13]](#endnote-14)^^ |
| **Alcohol consumption** | The risk of all-cause mortality, and of cancers specifically, rises with increasing levels of consumption, and the level of consumption that minimizes health loss is zero.^^[[14]](#endnote-15)^^ | People who recently quit drinking had an improvement in their quality of life that paralleled those that didn’t ever drink. ^^[[15]](#endnote-16)^^ |
| **Exercise** | Regular exercise significantly prolongs life^^[[16]](#endnote-17)^,^[[17]](#endnote-18)^,^[[18]](#endnote-19)^^ and disease-free survival^^[[19]](#endnote-20)^^ | …and enhances or improves quality of life,^^[[20]](#endnote-21)^^ opposes the deleterious effects of aging on muscle and muscle function by preventing the mitigating aging-related loss of muscle mass,^^[[21]](#endnote-22)^^  and plays an important role in preserving brain function, improving cognition, and provides protection against cognitive decline and dementia.^^[[22]](#endnote-23)^,^[[23]](#endnote-24)^,^[[24]](#endnote-25)^,^[[25]](#endnote-26)^^ |
| **Activity** | Sedentary life is associated with increased risk death^^[[26]](#endnote-27)^,^[[27]](#endnote-28)^,^^^[[28]](#endnote-29)^^ | …and increased risk of obesity,^26,28^ chronic disease, such as diabetes,^26^ cognitive impairment,^^[[29]](#endnote-30)^,^[[30]](#endnote-31)^^  physical disability or reduced physical function.^^[[31]](#endnote-32)^^ |
| **Sleep** | Chronic sleep deficiency associated with increased mortality^^[[32]](#endnote-33)^^ | …and associated with lower ratings of quality of life^^[[33]](#endnote-34)^^ |
| **Volunteering** | Volunteering associated with increased survival^^[[34]](#endnote-35)^,^[[35]](#endnote-36)^^ | …and with higher levels of happiness, mental well-being, and cognitive functioning.^^[[36]](#endnote-37)^,^[[37]](#endnote-38)^,^[[38]](#endnote-39)^^ |
| **Spirituality/Religion** | People with a more Spirituality/Religion beliefs and practices, have a higher chance of living longer^^[[39]](#endnote-40)^,^^^[[40]](#endnote-41)^,^[[41]](#endnote-42)^,^ | …and greater physical health.^^[[42]](#endnote-43)^^ |
| **Positive View of Self-Aging** | People with positive views on their aging are likely to live longer^^[[43]](#endnote-44)^^ | …and are more likely to report a better functional health over their future years.^^[[44]](#endnote-45)^^ |
| **Positive Mind Set** | People with a positive mind set more likely to live longer^^[[45]](#endnote-46)^,^[[46]](#endnote-47)^^ | …and to experience better mental and physical health and lower rates of chronic diseases.^^[[47]](#endnote-48)^,^[[48]](#endnote-49)^,^[[49]](#endnote-50)^,^[[50]](#endnote-51)^^ |
| **Social Networks** | People with stronger social networks are more likely to live longer^^[[51]](#endnote-52)^,^^^[[52]](#endnote-53)^,^[[53]](#endnote-54)^^ | …and more like to experience a greater quality of life^^[[54]](#endnote-55)^^ |
| **Social Supports** | People with stronger social supports are more likely to live longer^^[[55]](#endnote-56)^,^[[56]](#endnote-57)^,^[[57]](#endnote-58)^,^[[58]](#endnote-59)^^ | …and to enjoy better mental and physical health.^^[[59]](#endnote-60)^,^[[60]](#endnote-61)^,^[[61]](#endnote-62)^,^[[62]](#endnote-63)^,^[[63]](#endnote-64)^^ |
| **Social Activities** | A higher level of social activities associated with a survival advantage^^[[64]](#endnote-65)^,^[[65]](#endnote-66)^,^[[66]](#endnote-67)^,^[[67]](#endnote-68)^,^[[68]](#endnote-69)^,^[[69]](#endnote-70)^,^[[70]](#endnote-71)^^ | …and with greater physical function and well-being.^^[[71]](#endnote-72)^^ |
| **Life Purpose** | There is a strong association with ‘life purpose’ and longevity.^^[[72]](#endnote-73)^^ | People who find their own purpose are much more likely to enjoy an enhanced overall sense of physical well-being,^^[[73]](#endnote-74)^^ have greater resilience, increased global happiness and increased life satisfaction^^[[74]](#endnote-75)^,^^^[[75]](#endnote-76)^^ and better overall psychological well-being.^^[[76]](#endnote-77)^^ |
|  |  |  |
| ***Category 2*** |  |  |
| **Advance Medical Care Planning** |  | Going through some planning in advance for future medical treatments increases the likelihood of dying in location of choice and has significant impact on patient’s quality of life in remaining days, quality of death, and quality of life measures of substitute decision-makers/surrogates.^75^ |
| **Meditation** |  | Meditation/mindfulness associated with positive effects on quality of life.^^[[77]](#endnote-78)^^ |
| **Life-long learning** |  | Continuous participation in non-formal or formal life-long activities has been shown to have positive impact on a person’s overall health and well-being.^^[[78]](#endnote-79)^^ |
| **Leisure Participation** |  | Participation in leisure activities associated with improvement in overall well-being and life-satisfaction.^^[[79]](#endnote-80)^,^[[80]](#endnote-81)^^ |
| ***Practical Suggestions*** |  |  |
| **Regular Personal Planning** | nil | nil |
| **Legal Forms re: Substitute Decision-maker** | nil | nil |
| **Power of Attorney** | nil | nil |
| **Wills and Estate Planning** | nil | nil |
| **Financial Planning** | nil | nil |
| **Tax Planning** | nil | nil |
| **Small Business Succession Planning** | nil | nil |
| **Insurance** | nil | nil |
| **“Just in Case” Filing System** | nil | nil |
| **Caregiver Planning** | nil | nil |
| **Plan for Independent Living** | nil | nil |
| **Funeral Planning** | nil | nil |
| **Legacy Planning** | nil | nil |

**eTable 3 – Preparedness for the Future Questionnaire (Response Frequencies)**

|  | **All respondents (n=502)** |
| --- | --- |
| **1. What is your height?** | 79.9±21.6 (40.8, 215.0) |
| **2. What is your weight?** | 168.0±13.8 (100.0, 238.8) |
| **BMI** | 28.6±8.4 (10.3, 85.6) |
| **3. Do you eat a healthy diet?** |  |
| *Never* | 27 (5.4%) |
| *Rarely, on average, maybe one day a week or less* | 63 (12.5%) |
| *Occasionally, on average, maybe two days a week* | 88 (17.5%) |
| *Sometimes, on average, maybe 3 days a week* | 90 (17.9%) |
| *Frequently, on average, maybe 4 days a week* | 91 (18.1%) |
| *Usually, on average, maybe 5-6 days a week* | 79 (15.7%) |
| *Regularly, every day* | 61 (12.2%) |
| *Prefer not to say* | 3 (0.6%) |
| ***4. Do you smoke or currently use tobacco products?*** |  |
| *Yes, I regularly smoke or use tobacco products.* | 106 (21.1%) |
| *No, I do not currently smoke but have smoked or used tobacco products in the past.* | 150 (29.9%) |
| *No, I have never smoked or used tobacco products.* | 244 (48.6%) |
| *Prefer not to say* | 2 (0.4%) |
| ***5. On average how many alcoholic drinks do you consume a week?*** |  |
| *None, I do not drink alcohol or I do not drink on a weekly basis* | 252 (50.2%) |
| *1 to 7* | 192 (38.2%) |
| *8 to 14* | 28 (5.6%) |
| *15 or more* | 26 (5.2%) |
| *Prefer not to say* | 4 (0.8%) |
| **6. Do you exercise regularly?** |  |
| *Never* | 72 (14.3%) |
| *Rarely, on average, a few weeks a year or less* | 89 (17.7%) |
| *Occasionally, on average about 25% of the weeks in a year* | 96 (19.1%) |
| *Sometimes, on average about 50% of the weeks in a year* | 71 (14.1%) |
| *Frequently, on average about 75% of the weeks in a year* | 62 (12.4%) |
| *Usually, on average about 90% of the weeks in a year* | 28 (5.6%) |
| *Regularly, every week* | 79 (15.7%) |
| *Prefer not to say* | 5 (1.0%) |
| **7. During the past 7 days how would you rate your overall sleep quality?** |  |
| *Terrible* | 19 (3.8%) |
| *Fair* | 186 (37.1%) |
| *Poor* | 60 (12.0%) |
| *Good* | 191 (38.0%) |
| *Excellent* | 45 (9.0%) |
| *Prefer not to say* | 1 (0.2%) |
| **8. How often do you volunteer or serve others** |  |
| *Never* | 198 (39.4%) |
| *Rarely, maybe once or twice a year* | 103 (20.5%) |
| *Occasionally, maybe 3-4 times a year* | 56 (11.2%) |
| *Sometimes, maybe several times a year* | 50 (10.0%) |
| *Frequently, at least monthly* | 37 (7.4%) |
| *Usually, a couple times a month* | 21 (4.2%) |
| *Regularly, every week* | 32 (6.4%) |
| *Prefer not to say* | 5 (1.0%) |
| **9. a To what extent do you consider yourself to be religious?** |  |
| *Very religious* | 46 (9.2%) |
| *Moderately religious* | 101 (20.1%) |
| *Somewhat religious* | 125 (24.9%) |
| *Not at all religious* | 221 (44.0%) |
| *Prefer not to say* | 9 (1.8%) |
| **9. b To what extent do you consider yourself to be spiritual?** |  |
| *Very spiritual* | 75 (14.9%) |
| *Moderately spiritual* | 124 (24.7%) |
| *Somewhat spiritual* | 154 (30.7%) |
| *Not at all spiritual* | 139 (27.7%) |
| *Prefer not to say* | 10 (2.0%) |
| **10. How often do you do some form of meditation?** |  |
| *Never* | 259 (51.6%) |
| *Rarely, on average, maybe once a week or less* | 119 (23.7%) |
| *Occasionally, on average, maybe twice a week* | 36 (7.2%) |
| *Sometimes, on average, maybe 3 times a week* | 21 (4.2%) |
| *Frequently, on average, maybe 4 times a week* | 24 (4.8%) |
| *Usually, on average, maybe 5-6 times a week* | 8 (1.6%) |
| *Regularly, every day* | 27 (5.4%) |
| *Prefer not to say* | 8 (1.6%) |
| **11. How often do you take time to plan out your daily tasks or activities?** |  |
| *Never* | 42 (8.4%) |
| *Rarely, on average, maybe once a week or less* | 99 (19.7%) |
| *Occasionally, on average, maybe twice a week* | 103 (20.5%) |
| *Sometimes, on average, maybe 3 times a week* | 78 (15.5%) |
| *Frequently, on average, maybe 4 times a week* | 74 (14.7%) |
| *Usually, on average, maybe 5-6 times a week* | 36 (7.2%) |
| *Regularly, every day* | 65 (12.9%) |
| *Prefer not to say* | 5 (1.0%) |
| **12. Do you have an advance care plan living will advance directive or any form of written document** |  |
| *Yes* | 123 (24.5%) |
| *No* | 328 (65.3%) |
| *Not sure* | 51 (10.2%) |
| **12.b Does your plan or directive differentiate your wishes** | **n=123** |
| *No, my plan represents my wishes for care at the end of life only* | 24 (19.5%) |
| *Not sure what is the difference between serious illness and terminal illness or end of life* | 8 (6.5%) |
| *Yes, my plan includes my values and wishes for when I am seriously ill* | 91 (74.0%) |
| **13. Have you designated a Substitute Decision Maker** |  |
| *Yes* | 173 (34.5%) |
| *No* | 310 (61.8%) |
| *Not sure* | 19 (3.8%) |
| **13.b) Have you informed this person of their role in decision-making and educated them on your values and preferences?** |  |
| *Yes* | 155 (89.6%) |
| *No* | 14 (8.1%) |
| *Not sure* | 4 (2.3%) |
| **13.c) Have you signed legal papers to allow your named Substitute Decision-maker to function in this capacity?** |  |
| *Yes* | 123 (71.1%) |
| *No* | 41 (23.7%) |
| *Not sure* | 9 (5.2%) |
| **14. Do you have a will?** |  |
| *Yes* | 226 (45.0%) |
| *No* | 269 (53.6%) |
| *Not sure* | 7 (1.4%) |
| **14.b Have you reviewed your will recently?** |  |
| *Yes* | 135 (59.7%) |
| *No* | 90 (39.8%) |
| *Not sure* | 1 (0.4%) |
| **15. Do you have a Power of Attorney a legal document that names someone who will make other decisions related to personal care finance etc.** |  |
| *Yes* | 162 (32.3%) |
| *No* | 321 (63.9%) |
| *Not sure* | 19 (3.8%) |
| **16. When you think about your future as an older person do you feel positive or negative about this future?** |  |
| *Extremely positive* | 30 (6.0%) |
| *Very Positive* | 88 (17.5%) |
| *Somewhat Positive* | 147 (29.3%) |
| *Neutral, Neither Positive or Negative* | 120 (23.9%) |
| *Somewhat Negative* | 69 (13.7%) |
| *Very Negative* | 20 (4.0%) |
| *Extremely Negative* | 24 (4.8%) |
| *Prefer not to say* | 4 (0.8%) |
| **17. I am always optimistic about my future.** |  |
| *Maximally agree* | 27 (5.4%) |
| *Strongly agree* | 136 (27.1%) |
| *Moderately agree* | 218 (43.4%) |
| *Weakly agree* | 77 (15.3%) |
| *Do not agree at all* | 41 (8.2%) |
| *Prefer not to say* | 3 (0.6%) |
| **18. In my life I consistently work towards developing and maintaining strong social networks.** |  |
| *Maximally agree* | 43 (8.6%) |
| *Strongly agree* | 135 (26.9%) |
| *Moderately agree* | 186 (37.1%) |
| *Weakly agree* | 89 (17.7%) |
| *Do not agree at all* | 43 (8.6%) |
| *Prefer not to say* | 6 (1.2%) |
| **19. In my life I have strong social supports.** |  |
| *Maximally agree* | 66 (13.1%) |
| *Strongly agree* | 160 (31.9%) |
| *Moderately agree* | 171 (34.1%) |
| *Weakly agree* | 79 (15.7%) |
| *Do not agree at all* | 23 (4.6%) |
| *Prefer not to say* | 3 (0.6%) |
| **20. I believe that my life has a strong purpose.** |  |
| *Maximally agree* | 60 (12.0%) |
| *Strongly agree* | 151 (30.1%) |
| *Moderately agree* | 177 (35.3%) |
| *Weakly agree* | 71 (14.1%) |
| *Do not agree at all* | 40 (8.0%) |
| *Prefer not to say* | 3 (0.6%) |
| **21. I am a life-long learner.** |  |
| *Maximally agree* | 94 (18.7%) |
| *Strongly agree* | 177 (35.3%) |
| *Moderately agree* | 161 (32.1%) |
| *Weakly agree* | 51 (10.2%) |
| *Do not agree at all* | 17 (3.4%) |
| *Prefer not to say* | 2 (0.4%) |
| **22. Do you have a financial plan for your future?** |  |
| *Yes, I have a plan for at least 3 (or more) of the above components* | 140 (27.9%) |
| *Somewhat, I have a plan for 1-2 of the above components* | 163 (32.5%) |
| *No* | 167 (33.3%) |
| *Not sure* | 21 (4.2%) |
| *Prefer not to answer* | 11 (2.2%) |
| **23. Have you done tax planning for your future?** |  |
| *Yes* | 102 (20.3%) |
| *Partially* | 113 (22.5%) |
| *No* | 259 (51.6%) |
| *Not sure* | 16 (3.2%) |
| *Prefer not to answer* | 12 (2.4%) |
| **24. Are you a small business or farm business owner?** |  |
| *Yes* | 37 (7.4%) |
| *No* | 459 (91.4%) |
| *Not sure* | 3 (0.6%) |
| *Prefer not to answer* | 3 (0.6%) |
| **24.b) Do you have a business succession or transition plan?** | **n=37** |
| *Yes* | 12 (32.4%) |
| *No* | 19 (51.4%) |
| *Not sure what that is* | 5 (13.5%) |
| *Prefer not to answer* | 1 (2.7%) |
| **25. Do you have adequate insurance (life disability critical illness etc.) for your personal circumstances?** |  |
| *Yes* | 195 (38.8%) |
| *Somewhat* | 120 (23.9%) |
| *No* | 153 (30.5%) |
| *Not sure* | 19 (3.8%) |
| *Prefer not to answer* | 15 (3.0%) |
| **26. Do you have all your important files organized** |  |
| *Yes* | 176 (35.1%) |
| *Partially* | 165 (32.9%) |
| *No* | 130 (25.9%) |
| *Not sure* | 20 (4.0%) |
| *Prefer not to answer* | 11 (2.2%) |
| **27. Are one or more of your parents and parents-in-law still alive?** |  |
| *Yes* | 294 (58.6%) |
| *No* | 208 (41.4%) |
| **“Yes” to Question#27** | n=294 |
| *Their plans to maintain their living independently (if they so desire)* | 83 (28.2%) |
| *Their plans to remain in their home (or other setting of choice) as long as possible* | 103 (35.0%) |
| *Their values and preferences related to medical treatments offered near or at the end of life* | 87 (29.6%) |
| *The location of their important files* | 90 (30.6%) |
| **The remaining questions pertain to respondents 60 years or older (n=218)** | n=218 |
| **28. Do you have a plan to live independently in the setting of your choice as long as possible** |  |
| *Yes* | 134 (61.5%) |
| *Partially* | 49 (22.5%) |
| *No* | 28 (12.8%) |
| *Not sure* | 6 (2.8%) |
| *Prefer not to answer* | 1 (0.5%) |
| **29. I am very involved in social activities such as church volunteering clubs classes or other types of activities either formal or informal.** |  |
| *Maximally agree* | 9 (4.1%) |
| *Strongly agree* | 28 (12.8%) |
| *Moderately agree* | 52 (23.9%) |
| *Weakly agree* | 44 (20.2%) |
| *Do not agree at all* | 85 (39.0%) |
| **30. I have a very physically active lifestyle.** |  |
| *Maximally agree* | 18 (8.3%) |
| *Strongly agree* | 47 (21.6%) |
| *Moderately agree* | 78 (35.8%) |
| *Weakly agree* | 48 (22.0%) |
| *Do not agree at all* | 27 (12.4%) |
| **31. How frequently do you participate in leisure activities?** |  |
| *Never* | 5 (2.3%) |
| *Rarely, on average, maybe once a week or less* | 49 (22.5%) |
| *Occasionally, on average, maybe twice a week* | 39 (17.9%) |
| *Sometimes, on average, maybe 3 times a week* | 29 (13.3%) |
| *Frequently, on average, maybe 4 times a week* | 40 (18.3%) |
| *Usually, on average, maybe 5-6 times a week* | 17 (7.8%) |
| *Regularly, every day* | 39 (17.9%) |
| **32. Have you made plans for a funeral celebration of life or other event when you die?** |  |
| *Yes* | 65 (29.8%) |
| *No* | 143 (65.6%) |
| *Not sure* | 9 (4.1%) |
| *Prefer not to answer* | 1 (0.5%) |
| **33. Have you made a plan for the disposal of your body (to be buried cremated etc.)?** |  |
| *Yes* | 117 (53.7%) |
| *No* | 95 (43.6%) |
| *Not sure* | 5 (2.3%) |
| *Prefer not to answer* | 1 (0.5%) |
| **34. Do you have a legacy plan** |  |
| *Yes* | 39 (17.9%) |
| *Partially* | 45 (20.6%) |
| *No* | 133 (61.0%) |
| *Not sure* | 1 (0.5%) |

Values reported as n (%) or mean±standard deviation (min, max).

**eTable 4a – Results of Exploratory Factor Analysis: 7 factors model (among patients <60 years, n=284)**

| **Questions** | **Factor 1** | **Factor 2** | **Factor 3** | **Factor 4** | **Factor 5** | **Factor 6** | **Factor7** |
| --- | --- | --- | --- | --- | --- | --- | --- |
| **1,2 BMI** | -0.07 | 0.00 | 0.05 | -0.12 | -0.18 | 0.27 | -0.13 |
| **Q3.Healthy diet** | 0.03 | 0.17 | 0.08 | 0.06 | 0.12 | 0.57 | 0.12 |
| **Q4.Smoke** | -0.02 | -0.05 | 0.22 | -0.04 | 0.05 | 0.17 | 0.77 |
| **Q5.Alcoholic drinks** | -0.06 | 0.02 | -0.05 | -0.02 | 0.10 | -0.08 | 0.34 |
| **Q6.Exercise** | 0.01 | 0.13 | 0.12 | -0.03 | 0.12 | 0.60 | 0.07 |
| **Q7.Sleep quality** | -0.04 | 0.07 | 0.40 | 0.03 | 0.03 | 0.11 | 0.15 |
| **Q8. Serve others** | 0.06 | 0.19 | 0.13 | 0.04 | 0.45 | 0.14 | 0.06 |
| **Q9.Religious/spiritual** | -0.02 | 0.29 | 0.02 | 0.00 | 0.60 | 0.01 | 0.05 |
| **Q10.Meditation** | 0.02 | -0.01 | 0.05 | 0.02 | 0.58 | 0.18 | 0.12 |
| **Q11.Daily tasks** | 0.09 | 0.07 | 0.03 | 0.05 | 0.12 | 0.39 | -0.07 |
| **Q12 GoC** | 0.68 | 0.10 | 0.10 | 0.11 | 0.00 | -0.04 | -0.10 |
| **Q13.Substitute Decision Maker** | 0.67 | 0.15 | -0.02 | 0.11 | 0.05 | 0.04 | -0.01 |
| **Q14.Do you have a will** | 0.79 | -0.02 | 0.07 | 0.10 | 0.04 | 0.06 | -0.04 |
| **Q15.Power of Attorney** | 0.80 | 0.08 | -0.06 | 0.10 | 0.02 | 0.01 | 0.05 |
| **Q16.Feel about this future** | 0.12 | 0.45 | 0.64 | 0.08 | 0.17 | 0.07 | -0.09 |
| **Q17.Always optimistic** | 0.11 | 0.65 | 0.47 | 0.04 | 0.23 | 0.11 | -0.08 |
| **Q18.Strong social networks** | 0.16 | 0.68 | 0.12 | 0.20 | 0.12 | 0.19 | -0.03 |
| **Q19.Strong social supports** | 0.19 | 0.76 | -0.01 | 0.14 | -0.04 | 0.14 | 0.13 |
| **Q20.Strong purpose** | 0.03 | 0.71 | 0.23 | 0.03 | 0.27 | 0.10 | -0.06 |
| **Q21.Life-long learner** | 0.02 | 0.44 | 0.04 | 0.01 | 0.25 | 0.36 | -0.03 |
| **Q22.Financial plan** | 0.37 | 0.10 | 0.39 | 0.28 | 0.07 | 0.33 | 0.02 |
| **Q23.Tax planning** | 0.40 | 0.13 | 0.37 | 0.19 | -0.01 | 0.20 | -0.11 |
| **Q25.Adequate insurance** | 0.27 | 0.16 | 0.12 | 0.65 | 0.07 | -0.06 | -0.03 |
| **Q26.Important files organized** | 0.41 | 0.17 | 0.06 | 0.54 | -0.01 | 0.09 | -0.06 |

KMO=0.83

Bartlett's test: 156.4 (degrees of freedom=129), p=0.05

| Strong ≥ 0.7 |
| --- |
| Moderate 0.4 - < 0.7 |
| Weak 0.3 - < 0.4 |

| Positive Health Behavior |
| --- |
| Negative Health Behavior |
| Planning |
| Medico-Legal |
| Psychological Well-being |
| Enrichment |
| Late-life Planning |
| Social |

**eTable 4b – Results of Exploratory Factor Analysis: 7 factors model (among patients ≥60 years, n=218)**

| **Questions** | **Factor 1** | **Factor 2** | **Factor 3** | **Factor 4** | **Factor 5** | **Factor 6** | **Factor 7** |
| --- | --- | --- | --- | --- | --- | --- | --- |
| **1,2 BMI** | -0.20 | -0.03 | 0.16 | 0.09 | -0.03 | 0.13 | -0.30 |
| **Q3.Healthy diet** | 0.11 | 0.12 | 0.46 | 0.15 | 0.20 | 0.30 | 0.09 |
| **Q4.Smoke** | 0.14 | -0.06 | 0.09 | 0.14 | 0.13 | 0.06 | 0.27 |
| **Q5.Alcoholic drinks** | -0.10 | 0.06 | -0.02 | -0.06 | 0.09 | 0.03 | 0.48 |
| **Q6.Exercise** | 0.02 | 0.10 | 0.33 | 0.24 | 0.09 | 0.25 | -0.08 |
| **Q7.Sleep quality** | 0.06 | 0.20 | 0.41 | -0.13 | 0.01 | -0.14 | -0.14 |
| **Q8. Serve others** | 0.11 | 0.12 | 0.13 | 0.10 | 0.49 | 0.00 | 0.13 |
| **Q9.Religious/spiritual** | 0.02 | 0.19 | -0.09 | -0.08 | 0.57 | 0.31 | 0.22 |
| **Q10.Meditation** | -0.04 | 0.18 | -0.01 | -0.06 | 0.17 | 0.53 | 0.18 |
| **Q11.Daily tasks** | 0.10 | 0.02 | 0.07 | 0.10 | 0.00 | 0.55 | -0.11 |
| **Q12 GoC** | 0.73 | 0.04 | 0.05 | 0.06 | 0.03 | 0.06 | -0.07 |
| **Q13.Substitute Decision Maker** | 0.88 | 0.07 | 0.07 | 0.07 | 0.07 | 0.01 | 0.03 |
| **Q14.Do you have a will** | 0.58 | 0.03 | 0.05 | 0.28 | 0.05 | 0.06 | 0.15 |
| **Q15.Power of Attorney** | 0.71 | 0.11 | 0.04 | 0.26 | 0.08 | 0.01 | -0.03 |
| **Q16.Feel about this future** | 0.10 | 0.58 | 0.51 | 0.15 | -0.07 | 0.09 | 0.30 |
| **Q17.Always optimistic** | 0.02 | 0.74 | 0.33 | 0.08 | -0.21 | 0.12 | 0.17 |
| **Q18.Strong social networks** | 0.10 | 0.77 | -0.02 | 0.19 | 0.30 | -0.05 | -0.04 |
| **Q19.Strong social supports** | 0.09 | 0.69 | -0.04 | 0.22 | 0.22 | -0.04 | -0.08 |
| **Q20.Strong purpose** | 0.02 | 0.75 | 0.16 | 0.04 | 0.06 | 0.26 | 0.07 |
| **Q21.Life-long learner** | 0.10 | 0.41 | 0.14 | 0.01 | 0.29 | 0.19 | -0.07 |
| **Q22.Financial plan** | 0.08 | 0.09 | 0.14 | 0.67 | 0.08 | 0.14 | -0.11 |
| **Q23.Tax planning** | 0.18 | 0.03 | 0.12 | 0.69 | 0.11 | 0.03 | -0.05 |
| **Q25.Adequate insurance** | 0.13 | 0.17 | -0.08 | 0.42 | -0.07 | -0.06 | 0.03 |
| **Q26.Important files organized** | 0.38 | 0.21 | -0.12 | 0.52 | -0.06 | 0.06 | 0.13 |

Legend. Questions 24 and 27 were not included in this analysis because they were not answered by all respondents. Color coding of questions is based on the final domain and color coding of coefficients is based on the strength of association as shown below:

KMO=0.77

Bartlett's test: 184.3 (degrees of freedom=129)

p=0.001

| Strong ≥ 0.7 |
| --- |
| Moderate 0.4 - < 0.7 |
| Weak 0.3 - < 0.4 |

| Positive Health Behavior |
| --- |
| Negative Health Behavior |
| Planning |
| Medico-Legal |
| Psychological Well-being |
| Enrichment |
| Late-life Planning |
| Social |

**eTable 4c – Differences in Factor loadings between Participants <60 and 60 and Older**

| **Domain** |  | **Discrepancy between to subgroup EFAs** |
| --- | --- | --- |
| Medico-legal |  | No major differences |
| Social |  | No major differences |
| Psychological Well-being (PWB) Domain | EFA < 60 | No major differences |
|  | EFA ≥ 60 | ‘Always optimistic’ loads better on ‘Social’ domain compared to younger group |
| Planning | EFA < 60 | ‘Financial planning’ and ‘Tax planning’ load better on ‘PWB’ domain |
|  | EFA ≥ 60 | No major differences |
| Enrichment | EFA < 60 | ‘Life-long learner’ loads better on ‘Social’ domain |
|  | EFA ≥ 60 | ‘Meditation’ loads better on ‘Positive health’ |
| Positive Health | EFA < 60 | No major differences |
|  | EFA ≥ 60 | ‘Exercise’, ‘Maintaining BMI’, and ‘Healthy eating’ load better on ‘PWG’ domain. |
| Negative Health |  | No major differences |

Legend: Here we describe the differences in factor loadings when comparing the exploratory factor analysis in participants <60 years old compared to exploratory factor analysis in participants 60 or older.

**eTable 5 – Internal Reliability of the Prep FQ**

| **Questions/Domains** | | **n** | **Standardized Correlation with total** | **Standardized  Cronbach Alpha** | **McDonald Omega** |
| --- | --- | --- | --- | --- | --- |
| **Dom#1** | **Domain#1 Positive Health Behaviors (All participants)** | **502** |  | **0.50** | **0.53** |
| Q1+Q2 | Maintaining a normal BMI | 502 | 0.17 | 0.54 |  |
| Q3 | Eat a healthy diet | 502 | 0.34 | 0.39 |  |
| Q6 | Exercise regularly | 502 | 0.41 | 0.32 |  |
| Q11 | Take time to plan out your daily tasks or activities | 502 | 0.28 | 0.45 |  |
| **Dom#2** | **Domain#2 Negative Health Behaviors (All participants)** | **502** |  | **0.33** | **0.33** |
| Q4 | Smoke or currently use tobacco products | 502 | 0.20 | n/a |  |
| Q5 | Alcoholic drinks do you consume a week | 502 | 0.20 | n/a |  |
| **Dom#3** | **Domain#3 Planning (All participants without Q24b and Q27)** | **502** |  | **0.72** | **0.72** |
| Q22 | Have a financial plan for your future? | 502 | 0.54 | 0.63 |  |
| Q23 | Done tax planning for your future? | 502 | 0.54 | 0.63 |  |
| Q25 | Have adequate insurance | 502 | 0.41 | 0.71 |  |
| Q26 | Have all your important files organized | 502 | 0.63 | 0.64 |  |
| **Dom#3a** | **Domain#3 Planning (All participants with Q24b and Q27)** | **25** |  | **0.88** | **0.89** |
| Q22 | Have a financial plan for your future? | 25 | 0.80 | 0.83 |  |
| Q23 | Done tax planning for your future? | 25 | 0.81 | 0.83 |  |
| Q24b | Have a business succession or transition plan? | 25 | 0.76 | 0.84 |  |
| Q25 | Have adequate insurance | 25 | 0.70 | 0.85 |  |
| Q26 | Have all your important files organized | 25 | 0.68 | 0.86 |  |
| Q27b | Helped your parents/in-laws with their planning | 25 | 0.37 | 0.91 |  |
| **Dom#3b** | **Domain#3 Planning (All participants without Q24b)** | **294** |  | **0.68** | **0.69** |
| Q22 | Have a financial plan for your future? | 294 | 0.56 | 0.57 |  |
| Q23 | Done tax planning for your future? | 294 | 0.52 | 0.59 |  |
| Q25 | Have adequate insurance | 294 | 0.39 | 0.64 |  |
| Q26 | Have all your important files organized | 294 | 0.52 | 0.59 |  |
| Q27b | Helped your parents/in-laws with their planning | 294 | 0.19 | 0.73 |  |
| **Dom#3c** | **Domain#3 Planning (All participants without Q27)** | **37** |  | **0.84** | **0.84** |
| Q22 | Have a financial plan for your future? | 37 | 0.63 | 0.81 |  |
| Q23 | Done tax planning for your future? | 37 | 0.78 | 0.77 |  |
| Q24b | Have a business succession or transition plan? | 37 | 0.66 | 0.80 |  |
| Q25 | Have adequate insurance | 37 | 0.59 | 0.82 |  |
| Q26 | Have all your important files organized | 37 | 0.57 | 0.83 |  |
| **Dom#4** | **Domain#4 Medico-Legal (All participants)** | **502** |  | **0.85** | **0.86** |
| Q12 | Filled out an Advance Care Plan | 502 | 0.66 | 0.83 |  |
| Q13 | Designated a Substitute Decision Maker | 502 | 0.72 | 0.80 |  |
| Q14 | Have a will | 502 | 0.68 | 0.82 |  |
| Q15 | Have a Power of Attorney | 502 | 0.73 | 0.80 |  |
| **Dom#5** | **Domain#5 Psychological Well-being (All participants)** | **502** |  | **0.67** | **0.72** |
| Q7 | Rate your overall sleep quality | 502 | 0.28 | 0.82 |  |
| Q16 | Positive views on self as an older person | 502 | 0.62 | 0.39 |  |
| Q17 | Always optimistic about my future. | 502 | 0.59 | 0.43 |  |
| **Dom#6** | **Domain#6 Enrichment (All participants)** | **502** |  | **0.60** | **0.61** |
| Q8 | Volunteer or serve others | 502 | 0.37 | 0.54 |  |
| Q9 | Consider yourself to be religious/spiritual? | 502 | 0.46 | 0.47 |  |
| Q10 | How often do you do some form of meditation | 502 | 0.36 | 0.55 |  |
| Q21 | I am a life-long learner. | 502 | 0.34 | 0.56 |  |
| **Dom#7** | **Domain#7 –Social (All participants)** | **502** |  | **0.81** | **0.81** |
| Q18 | Developing and maintaining strong social networks. | 502 | 0.69 | 0.70 |  |
| Q19 | Have strong social supports. | 502 | 0.67 | 0.71 |  |
| Q20 | Believe that my life has a strong purpose | 502 | 0.60 | 0.79 |  |
| **Dom#8** | **Domain#8 -Late-life Planning (Participants > 60)** | **218** |  | **0.61** | **0.58** |
| Q28 | Plan to live independently | 218 | 0.28 | 0.58 |  |
| Q29 | Very involved in social activities | 218 | 0.35 | 0.56 |  |
| Q30 | Have a very physically active lifestyle | 218 | 0.27 | 0.59 |  |
| Q31 | Participate in leisure activities | 218 | 0.20 | 0.61 |  |
| Q32 | Made plans for a funeral or celebration of life | 218 | 0.45 | 0.52 |  |
| Q33 | Made a plan for the disposal of your body | 218 | 0.39 | 0.55 |  |
| Q34 | Have a legacy plan | 218 | 0.32 | 0.57 |  |

Legend: As not all participants answered all questions, results are reported separately for those age <60 and > 60 years where applicable. Questions 24 and 27 were not answered by all respondents resulting in 4 different evaluations of the reliability of domain #3. The Cronbach alpha’s listed beside the individual items are the alphas that would results if that item was removed from the domain. When removal of an item increases the Cronbach alpha’s then this suggests that the item may not belong to the same latent construct measured by the other items. A low correlation between an item and the total of the other items within the same domain, shown in second from right hand side, also suggest that the item is not measuring the same construct as the other items in the domain.

**eTable 6 – Internal reliability of Other Questionnaires**

| **Questions/Domains** | **All respondents** | | **Respondents age ≥ 60** | | **Respondents**  **Age <60** | |
| --- | --- | --- | --- | --- | --- | --- |
|  | **n** | **Standardized  Cronbach Alpha** | **n** | **Standardized  Cronbach Alpha** | **n** | **Standardized  Cronbach Alpha** |
| **GRLS** | **n/a** | **n/a** | **n/a** | **n/a** | **n/a** | **n/a** |
| **Physical Component Summary Score (PCS)** | 502 | 0.76 | 218 | 0.74 | 284 | 0.78 |
| **Mental Component Summary Score (MCS)** | 502 | 0.78 | 218 | 0.83 | 284 | 0.74 |
| **PWB overall score** | 502 | 0.94 | 218 | 0.94 | 284 | 0.94 |
| **PWB Autonomy** | 502 | 0.77 | 218 | 0.77 | 284 | 0.75 |
| **PWB Environmental mastery** | 502 | 0.56 | 218 | 0.49 | 284 | 0.57 |
| **PWB Personal Growth** | 502 | 0.76 | 218 | 0.79 | 284 | 0.74 |
| **PWB Positive Relations** | 502 | 0.82 | 218 | 0.84 | 284 | 0.79 |
| **PWB Purpose in life** | 502 | 0.75 | 218 | 0.76 | 284 | 0.74 |
| **PWB Self-acceptance** | 502 | 0.89 | 218 | 0.90 | 284 | 0.87 |

**eTable 7 – Correlations Amongst Prep FQ and Other Questionnaires**

| **Domains of Prep FQ** | **GRLS** | **Physical Component Summary Score (PCS)** | **Mental Component Summary Score (MCS)** | **PWB overall score** | **PWB**  **Autonomy** | **PWB**  **Environmental mastery** | **PWB**  **Personal Growth** | **PWB**  **Positive Relations** | **PWB**  **Purpose in life** | **PWB**  **Self-acceptance** |
| --- | --- | --- | --- | --- | --- | --- | --- | --- | --- | --- |
| **1. Positive Health Behaviors** | **0.20** <.0001 | **0.29** <.0001 | **0.16** 0.0002 | **0.24** <.0001 | **0.13** 0.004 | **0.20** <.0001 | **0.29** <.0001 | **0.18** <.0001 | **0.24** <.0001 | **0.19** <.0001 |
| **2. Negative Health Behaviors** | **0.03** 0.46 | **0.002** 0.96 | **0.05** 0.30 | **-0.05** 0.23 | **-0.10** 0.03 | **-0.11** 0.01 | **-0.02** 0.66 | **-0.07** 0.1366 | **0.06** 0.17 | **-0.05** 0.29 |
| **3. Planning** | **0.32** <.0001 | **0.01** 0.75 | **0.27** <.0001 | **0.37** <.0001 | **0.20**  <.0001 | **0.44** <.0001 | **0.22** <.0001 | **0.35** <.0001 | **0.31** <.0001 | **0.34** <.0001 |
| **4. Medico-Legal** | **0.15** 0.0005 | **-0.11** 0.01 | **0.12** 0.010 | **0.19** <.0001 | **0.13** 0.003 | **0.21** <.0001 | **0.08** 0.06 | **0.21** <.0001 | **0.13** 0.005 | **0.19** <.0001 |
| **5. Psychological Well-being** | **0.68** <.0001 | **0.22**  <.0001 | **0.51** <.0001 | **0.60** <.0001 | **0.32** <.0001 | **0.52** <.0001 | **0.46** <.0001 | **0.47** <.0001 | **0.55** <.0001 | **0.63** <.0001 |
| **6. Enrichment** | **0.23** <.0001 | **-0.06** 0.16 | **0.08** 0.06 | **0.32** <.0001 | **0.20** <.0001 | **0.23** <.0001 | **0.37** <.0001 | **0.27** <.0001 | **0.28** <.0001 | **0.26** <.0001 |
| **7. Late-life Planning** | **0.17** 0.01 | **-0.05** 0.46 | **0.02** 0.79 | **0.24** 0.0003 | **0.14** 0.03 | **0.28** <.0001 | **0.17** 0.01 | **0.23** 0.0007 | **0.16** 0.02 | **0.21** 0.0016 |
| **8. Social** | **0.48** <.0001 | **0.12** 0.007 | **0.20** <.0001 | **0.46** <.0001 | **0.09** 0.04 | **0.39** <.0001 | **0.38** <.0001 | **0.55** <.0001 | **0.42** <.0001 | **0.42** <.0001 |
| **Overall Prep FQ score** | **0.53** <.0001 | **0.17** <.0001 | **0.35** <.0001 | **0.54** <.0001 | **0.24** <.0001 | **0.44** <.0001 | **0.48** <.0001 | **0.47** <.0001 | **0.51** <.0001 | **0.49** <.0001 |

Legend: Correlations (bolded) and associated p values. Prep FQ- Preparedness for the Future Questionnaire; GRLS- Global Rating of Life Satisfaction; PWB- Psychological Well-being.

References

1. Berrington de Gonzalez A, Hartge P, Cerhan JR, Flint AJ, Hannan L, MacInnis RJ, et al. Body-mass index and mortality among 1.46 million white adults. N Engl J Med. 2010;363:2211-9. [↑](#endnote-ref-2)
2. Nyberg ST, Singh-Manoux A, Pentti J, Madsen IEH, Sabia S, Alfredsson L, et al. Association of Healthy Lifestyle With Years Lived Without Major Chronic Diseases. JAMA Intern Med. 2020;180:760-768. [↑](#endnote-ref-3)
3. Pedditzi E, Peters R, Beckett N. The risk of overweight/obesity in mid-life and late life for the development of dementia: a systematic review and meta-analysis of longitudinal studies. Age Ageing. 2016;45:14-21. [↑](#endnote-ref-4)
4. Pantalone KM, Hobbs TM, Chagin KM, Kong SX, Wells BJ, Kattan MW, et al. Prevalence and recognition of obesity and its associated comorbidities: cross-sectional analysis of electronic health record data from a large US integrated health system. BMJ Open. 2017;7:e017583. doi: 10.1136/bmjopen-2017-017583. [↑](#endnote-ref-5)
5. Rietman ML, van der A DL, van Oostrom SH, Picavet HSJ, Dollé MET, van Steeg H, et al. The Association between BMI and Different Frailty Domains: A U-Shaped Curve? J Nutr Health Aging. 2018;22:8-15. [↑](#endnote-ref-6)
6. Callaghan BC, Reynolds EL, Banerjee M, Chant E, Villegas-Umana E, Gardner TW, et al. The Prevalence and Determinants of Cognitive Deficits and Traditional Diabetic Complications in the Severely Obese. Diabetes Care. 2020;43:683-690 [↑](#endnote-ref-7)
7. Berry KM, Neogi T, Baker JF, Collins JM, Waggoner JR, Hsiao CW, et al. Obesity Progression between Young Adulthood and Midlife and Incident Arthritis: A Retrospective Cohort Study of US Adults. Arthritis Care Res (Hoboken). 2020; doi: 10.1002/acr.24252. [↑](#endnote-ref-8)
8. Yu JT, Xu W, Tan CC, Andrieu S, Suckling J, Evangelou E, et al. Evidence-based prevention of Alzheimer's disease: systematic review and meta-analysis of 243 observational prospective studies and 153 randomised controlled trials. J Neurol Neurosurg Psychiatry. 2020; doi: 10.1136/jnnp-2019-321913.  [↑](#endnote-ref-9)
9. Hu EA, Steffen LM, Coresh J, Appel LJ, Rebholz CM. Adherence to the Healthy Eating Index-2015 and Other Dietary Patterns May Reduce Risk of Cardiovascular Disease, Cardiovascular Mortality, and All-Cause Mortality. J Nutr. 2020;150:312-321. [↑](#endnote-ref-10)
10. Hengeveld LM, Wijnhoven HAH, Olthof MR, Brouwer IA, Simonsick EM, Kritchevsky SB, et al. Prospective Associations of Diet Quality With Incident Frailty in Older Adults: The Health, Aging, and Body Composition Study. J Am Geriatr Soc. 2019;67:1835-1842. [↑](#endnote-ref-11)
11. U.S. Department of Health and Human Services. The Health Consequences of Smoking—50 Years of Progress: A Report of the Surgeon General. Atlanta: U.S. Department of Health and Human Services, Centers for Disease Control and Prevention, National Center for Chronic Disease Prevention and Health Promotion, Office on Smoking and Health. 2014. <https://www.cdc.gov/tobacco/data_statistics/sgr/50th-anniversary/index.htm>. Accessed 20 April 2017. [↑](#endnote-ref-12)
12. U.S. Department of Health and Human Services. How Tobacco Smoke Causes Disease: What It Means to You. Atlanta: U.S. Department of Health and Human Services, Centers for Disease Control and Prevention, National Center for Chronic Disease Prevention and Health Promotion, Office on Smoking and Health. 2010. <https://www.cdc.gov/tobacco/data_statistics/sgr/2010/consumer_booklet/index.htm>. Accessed 20 April 2017. [↑](#endnote-ref-13)
13. Swayambunathan J, Dasgupta A, Rosenberg PS, Hannan MT, Kiel DP, Bhattacharyya T. Incidence of Hip Fracture Over 4 Decades in the Framingham Heart Study. JAMA Intern Med. 2020;180:1–7. [↑](#endnote-ref-14)
14. GBD 2016 Alcohol Collaborators. Alcohol use and burden for 195 countries and territories, 1990-2016: a systematic analysis for the Global Burden of Disease Study 2016. Lancet. 2018;392:1015-1035. [↑](#endnote-ref-15)
15. Yao XI, Ni MY, Cheung F, Wu JT, Schooling CM, Leung GM, et al. Change in moderate alcohol consumption and quality of life: evidence from 2 population-based cohorts. CMAJ. 2019;191:E753-E760. [↑](#endnote-ref-16)
16. Zhao M, Veeranki SP, Magnussen CG, Xi B. Recommended physical activity and all cause and cause specific mortality in US adults: prospective cohort study. BMJ. 2020;370:m2031. [↑](#endnote-ref-17)
17. Li Y, Pan A, Wang DD, Liu X, Dhana K, Franco OH, Kaptoge S, Di Angelantonio E, Stampfer M, Willett WC, Hu FB. Impact of Healthy Lifestyle Factors on Life Expectancies in the US Population. Circulation. 2018;138:345-355. [↑](#endnote-ref-18)
18. Lee IM, Shiroma EJ, Kamada M, Bassett DR, Matthews CE, Buring JE. Association of Step Volume and Intensity With All-Cause Mortality in Older Women. JAMA Intern Med. 2019;179:1105–12. [↑](#endnote-ref-19)
19. Li Y, Schoufour J, Wang DD, Dhana K, Pan A, Liu X, et al. Healthy lifestyle and life expectancy free of cancer, cardiovascular disease, and type 2 diabetes: prospective cohort study. BMJ. 2020;368:l6669. [↑](#endnote-ref-20)
20. Ballin M, Lundberg E, Sörlén N, Nordström P, Hult A, Nordström A. Effects of interval training on quality of life and cardiometabolic risk markers in older adults: a randomized controlled trial. Clin Interv Aging. 2019;14:1589-1599. [↑](#endnote-ref-21)
21. Cartee GD, Hepple RT, Bamman MM, Zierath JR. Exercise Promotes Healthy Aging of Skeletal Muscle. Cell Metab. 2016;23:1034-1047.  [↑](#endnote-ref-22)
22. Panza GA, Taylor BA, MacDonald HV, Johnson BT, Zaleski AL, Livingston J, Thompson PD, Pescatello LS. Can Exercise Improve Cognitive Symptoms of Alzheimer's Disease? J Am Geriatr Soc. 2018;66:487-495. [↑](#endnote-ref-23)
23. Hamer M, Chida Y. Physical activity and risk of neurodegenerative disease: a systematic review of prospective evidence. Psychol Med. 2009;39:3-11.  [↑](#endnote-ref-24)
24. Guadagni V, Drogos LL, Tyndall AV, Davenport MH, Anderson TJ, Eskes GA, et al. Aerobic exercise improves cognition and cerebrovascular regulation in older adults. Neurology. 2020;94:e2245-e2257. [↑](#endnote-ref-25)
25. Yu JT, Xu W, Tan CC, Andrieu S, Suckling J, Evangelou E, et al. Evidence-based prevention of Alzheimer's disease: systematic review and meta-analysis of 243 observational prospective studies and 153 randomised controlled trials. J Neurol Neurosurg Psychiatry. 2020; doi: 10.1136/jnnp-2019-321913. [↑](#endnote-ref-26)
26. Copeland JL, Ashe MC, Biddle SJ, Brown WJ, Buman MP, Chastin S, et al. Sedentary time in older adults: a critical review of measurement, associations with health, and interventions. Br J Sports Med. 2017;51:1539. [↑](#endnote-ref-27)
27. Biswas A, Oh PI, Faulkner GE, Bajaj RR, Silver MA, Mitchell MS, et al. Sedentary time and its association with risk for disease incidence, mortality, and hospitalization in adults: a systematic review and meta-analysis. Ann Intern Med. 2015;162:123-32. [↑](#endnote-ref-28)
28. Ku PW, Steptoe A, Liao Y, Hsueh MC, Chen LJ. A cut-off of daily sedentary time and all-cause mortality in adults: a meta-regression analysis involving more than 1 million participants. BMC Med. 2018;16:74. [↑](#endnote-ref-29)
29. Rosenberg DE, Bellettiere J, Gardiner PA, Villarreal VN, Crist K, Kerr J. Independent Associations Between Sedentary Behaviors and Mental, Cognitive, Physical, and Functional Health Among Older Adults in Retirement Communities. J Gerontol A Biol Sci Med Sci. 2016;71:78-83. [↑](#endnote-ref-30)
30. Falck RS, Davis JC, Liu-Ambrose T. What is the association between sedentary behaviour and cognitive function? A systematic review. Br J Sports Med. 2017;51:800-811. [↑](#endnote-ref-31)
31. Dunlop DD, Song J, Arnston EK, Semanik PA, Lee J, Chang RW, et al. Sedentary time in US older adults associated with disability in activities of daily living independent of physical activity. J Phys Act Health. 2015;12:93-101.  [↑](#endnote-ref-32)
32. Itani O, Jike M, Watanabe N, Kaneita Y. Short sleep duration and health outcomes: a systematic review, meta-analysis, and meta-regression. Sleep Med. 2017;32:246-256. [↑](#endnote-ref-33)
33. Medic G, Wille M, Hemels ME. Short- and long-term health consequences of sleep disruption. Nat Sci Sleep. 2017;9:151-161.  [↑](#endnote-ref-34)
34. Kim ES, Whillans AV, Lee MT, Chen Y, VanderWeele TJ. Volunteering and Subsequent Health and Well-Being in Older Adults: An Outcome-Wide Longitudinal Approach. Am J Prev Med. 2020;59:176-186. [↑](#endnote-ref-35)
35. Okun MA, Yeung EW, Brown S. Volunteering by older adults and risk of mortality: a meta-analysis. Psychol Aging. 2013;28:564-77. [↑](#endnote-ref-36)
36. Proulx CM, Curl AL, Ermer AE. Longitudinal Associations Between Formal Volunteering and Cognitive Functioning. J Gerontol B Psychol Sci Soc Sci. 2018;73:522-531. [↑](#endnote-ref-37)
37. Dulin PL, Gavala J, Stephens C, Kostick M, McDonald J. Volunteering predicts happiness among older Māori and non-Māori in the New Zealand health, work, and retirement longitudinal study. Aging Ment Health. 2012;16:617-24. [↑](#endnote-ref-38)
38. Pitkala KH, Routasalo P, Kautiainen H, Sintonen H, Tilvis RS. Effects of socially stimulating group intervention on lonely, older people's cognition: a randomized, controlled trial. Am J Geriatr Psychiatry. 2011;19:654-63. [↑](#endnote-ref-39)
39. Steinhauser KE, Fitchett G, Handzo GF, Johnson KS, Koenig HG, Pargament KI, et al. State of the Science of Spirituality and Palliative Care Research Part I: Definitions, Measurement, and Outcomes. J Pain Symptom Manage. 2017;54:428-440. [↑](#endnote-ref-40)
40. Mueller PS, Plevak DJ, Rummans TA. Religious involvement, spirituality, and medicine: implications for clinical practice. Mayo Clin Proc. 2001;76:1225-35. [↑](#endnote-ref-41)
41. Koenig HG. Religion, spirituality, and health: the research and clinical implications. ISRN Psychiatry. 2012;2012:278730. [↑](#endnote-ref-42)
42. Czekierda K, Banik A, Park CL, Luszczynska A. Meaning in life and physical health: systematic review and meta-analysis. Health Psychol Rev. 2017;11:387-418.  [↑](#endnote-ref-43)
43. Levy BR, Slade MD, Kunkel SR, Kasl SV. Longevity increased by positive self-perceptions of aging. J Pers Soc Psychol. 2002;83:261-70.

    [↑](#endnote-ref-44)
44. Levy BR, Slade MD, Kasl SV. Longitudinal benefit of positive self-perceptions of aging on functional health. J Gerontol B Psychol Sci Soc Sci. 2002;57:P409-17. [↑](#endnote-ref-45)
45. Steptoe A, Wright C, Kunz-Ebrecht SR, Iliffe S. Dispositional optimism and health behaviour in community-dwelling older people: associations with healthy ageing. Br J Health Psychol. 2006;11:71-84. [↑](#endnote-ref-46)
46. Kim ES, Hagan KA, Grodstein F, DeMeo DL, De Vivo I, Kubzansky LD. Optimism and Cause-Specific Mortality: A Prospective Cohort Study. Am J Epidemiol. 2017;185:21-29. [↑](#endnote-ref-47)
47. Peterson P, Park N, Kim ES. Can optimism decrease the risk of illness and disease among the elderly? Aging Health. 2012;8:5-8. [↑](#endnote-ref-48)
48. Rasmussen HN, Scheier MF, Greenhouse JB. Optimism and physical health: a meta-analytic review. Ann Behav Med. 2009;37:239-56. [↑](#endnote-ref-49)
49. Orom H, Nelson CJ, Underwood W 3rd, Homish DL, Kapoor DA. Factors associated with emotional distress in newly diagnosed prostate cancer patients. Psychooncology. 2015;24:1416-22. [↑](#endnote-ref-50)
50. Litt MD, Tennen H, Affleck G, Klock S. Coping and cognitive factors in adaptation to in vitro fertilization failure. J Behav Med. 1992;15:171-87. [↑](#endnote-ref-51)
51. Golden J, Conroy RM, Bruce I, Denihan A, Greene E, Kirby M, et al. Loneliness, social support networks, mood and wellbeing in community-dwelling elderly. Int J Geriatr Psychiatry. 2009;24:694-700. [↑](#endnote-ref-52)
52. Courtin E, Knapp M. Social isolation, loneliness and health in old age: a scoping review. Health Soc Care Community. 2017;25:799-812. [↑](#endnote-ref-53)
53. Iwasaki M, Otani T, Sunaga R, Miyazaki H, Xiao L, Wang N, et al. Social networks and mortality based on the Komo-Ise cohort study in Japan. Int J Epidemiol. 2002;31:1208-18. [↑](#endnote-ref-54)
54. Eklund M, Hansson L. Social network among people with persistent mental illness: associations with sociodemographic, clinical and health-related factors. Int J Soc Psychiatry. 2007;53:293-305. [↑](#endnote-ref-55)
55. André-Petersson L, Hedblad B, Janzon L, Ostergren PO. Social support and behavior in a stressful situation in relation to myocardial infarction and mortality: who is at risk? Results from prospective cohort study "Men born in 1914," Malmö, Sweden. Int J Behav Med. 2006;13:340-7. [↑](#endnote-ref-56)
56. Zhang X, Norris SL, Gregg EW, Beckles G. Social support and mortality among older persons with diabetes. Diabetes Educ. 2007;33:273-81. [↑](#endnote-ref-57)
57. Holt-Lunstad J, Smith TB, Layton JB. Social relationships and mortality risk: a meta-analytic review. PLoS Med. 2010;7:e1000316. doi: 10.1371/journal.pmed.1000316. [↑](#endnote-ref-58)
58. Penninx BW, van Tilburg T, Kriegsman DM, Deeg DJ, Boeke AJ, van Eijk JT. Effects of social support and personal coping resources on mortality in older age: the Longitudinal Aging Study Amsterdam. Am J Epidemiol. 1997;146:510-9. [↑](#endnote-ref-59)
59. Dalgard OS, Bjørk S, Tambs K. Social support, negative life events and mental health. Br J Psychiatry. 1995;166:29-34. [↑](#endnote-ref-60)
60. Mathiesen KS, Tambs K, Dalgard OS. The influence of social class, strain and social support on symptoms of anxiety and depression in mothers of toddlers. Soc Psychiatry Psychiatr Epidemiol. 1999;34:61-72. [↑](#endnote-ref-61)
61. Johnson JV, Hall EM. Job strain, work place social support, and cardiovascular disease: a cross-sectional study of a random sample of the Swedish working population. Am J Public Health. 1988;78:1336-42. [↑](#endnote-ref-62)
62. Berkman LF, Leo-Summers L, Horwitz RI. Emotional support and survival after myocardial infarction. A prospective, population-based study of the elderly. Ann Intern Med. 1992;117:1003-9. [↑](#endnote-ref-63)
63. Seeman TE. Social ties and health: the benefits of social integration. Ann Epidemiol. 1996;6:442-51. [↑](#endnote-ref-64)
64. House JS, Robbins C, Metzner HL. The association of social relationships and activities with mortality: prospective evidence from the Tecumseh Community Health Study. Am J Epidemiol. 1982;116:123-40. [↑](#endnote-ref-65)
65. Haak M, Löfqvist C, Ullén S, Horstmann V, Iwarsson S. The influence of participation on mortality in very old age among community-living people in Sweden. Aging Clin Exp Res. 2019;31:265-271. [↑](#endnote-ref-66)
66. Jylhä M, Aro S. Social ties and survival among the elderly in Tampere, Finland. Int J Epidemiol. 1989;18:158-64. [↑](#endnote-ref-67)
67. Ho SC. Health and social predictors of mortality in an elderly Chinese cohort. Am J Epidemiol. 1991;133:907-21. [↑](#endnote-ref-68)
68. Walter-Ginzburg A, Blumstein T, Chetrit A, Modan B. Social factors and mortality in the old-old in Israel: the CALAS study. J Gerontol B Psychol Sci Soc Sci. 2002;57:S308-18. [↑](#endnote-ref-69)
69. Sabin EP. Social Relationships and Mortality Among the Elderly. J Appl Gerontol. 1993;12:44-60. [↑](#endnote-ref-70)
70. Glass TA, de Leon CM, Marottoli RA, Berkman LF. Population based study of social and productive activities as predictors of survival among elderly Americans. BMJ. 1999 Aug 21;319(7208):478-83. [↑](#endnote-ref-71)
71. Cherry KE, Walker EJ, Brown JS, Volaufova J, LaMotte LR, Welsh DA, et al. Social engagement and health in younger, older, and oldest-old adults in the Louisiana Healthy Aging Study. J Appl Gerontol. 2013;32:51-75. [↑](#endnote-ref-72)
72. Alimujiang A, Wiensch A, Boss J, Fleischer NL, Mondul AM, McLean K, et al. Association Between Life Purpose and Mortality Among US Adults Older Than 50 Years. JAMA Netw Open. 2019;2:e194270. [↑](#endnote-ref-73)
73. Bonebright CA, Clay DL, Ankenmann RD. The relationship of workaholism with work–life conflict, life satisfaction, and purpose in life. J Couns Psychol. 2000;47:469-477. [↑](#endnote-ref-74)
74. Debats DL, Van der Lubbe PM, Wezeman FR. On the psychometric properties of the Life Regard Index (LRI): A measure of meaningful life. Pers Individ Dif. 1993;14:337-345. [↑](#endnote-ref-75)
75. Kim ES, Strecher VJ, Ryff CD. Purpose in life and use of preventive health care services. Proc Natl Acad Sci U S A. 2014;111:16331-6. [↑](#endnote-ref-76)
76. Cohen R, Bavishi C, Rozanski A. Purpose in Life and Its Relationship to All-Cause Mortality and Cardiovascular Events: A Meta-Analysis. Psychosom Med. 2016;78:122-33. [↑](#endnote-ref-77)
77. Davis DM, Hayes JA. What are the benefits of mindfulness. Monit Psychol. 2012;43:64. [↑](#endnote-ref-78)
78. Dolan P, Fujiwara D, Metcalfe R. Review and Update of Research into the Wider Benefits of Adult Learning. BIS Research Paper Number 90, Department for Business, Innovation & Skills, London. 2012. [↑](#endnote-ref-79)
79. Paggi ME, Jopp D, Hertzog C. The Importance of Leisure Activities in the Relationship between Physical Health and Well-Being in a Life Span Sample. Gerontology. 2016;62:450-8. [↑](#endnote-ref-80)
80. Han JS, Patterson J. An Analysis of the Influence That Leisure Experiences Have on a Person's Mood State, Health and Wellbeing. Ann Leis Res. 2007;10:328-351. [↑](#endnote-ref-81)
